# RNA-seq analysis of apical meristem reveals integrative regulatory network of ROS and chilling potentially related to flowering in *Litchi chinensis*

**DOI:** 10.1038/s41598-017-10742-y

**Published:** 2017-08-31

**Authors:** Xingyu Lu, Jingjing Li, Houbin Chen, Jiaqi Hu, Pengxu Liu, Biyan Zhou

**Affiliations:** College of Horticulture, South China Agricultural University, Guangzhou, 510642 China

## Abstract

Litchi is an important woody fruit tree. Floral initiation in litchi is triggered by low temperatures. However, defective flowering is a major challenge for litchi production in times of climate change and global warming. Previous studies have shown that the reactive oxygen species (ROS) generated by methyl viologen dichloride hydrate (MV) promotes flowering. In this study, potted trees were transferred to growth chambers for low-temperature (LT), medium-temperature (MT), and high-temperature (HT) treatments. Trees at MT were subjected to ROS treatment to promote flowering, and those at LT were induced to flower. RNA-sequencing was applied to obtain a global transcriptome of the apical meristem and reveal potential gene networks controlling the transformation from vegetative meristems (VM) into inflorescence meristems (IM). We assembled 73,117 unigenes with a mean size of 790 bp and 11741 unigenes were identified as both chilling and ROS responsive genes (CRRGs), of which 48 were identified as flowering-related CRRGs, 59 were plant hormone signal transduction CRRGs, and 146 were plant hormone biosynthesis-related CRRGs. Genes co-expression network analysis indicated inner relationships, suggesting that ROS and chilling promotes the VM to IM transition through a regulatory gene network of transcription factors, hormones, and flowering regulators.

## Introduction

Litchi is an evergreen fruit tree in southern Asia. Floral initiation in litchi is triggered by low temperatures and enhanced by drought in autumn and winter^1, 2^. Warm winters, which potentially result from global warming, lead to poor litchi flowering. It is important to understand the genetics of flowering to find ways to bypass chilling. In the model plant Arabidopsis, the genetics of the flowering network have been well studied. CO is a direct activator of *FLOWERING LOCUS T* (*FT*)^3^, which encodes a long seeking florigen protein that migrates from the leaves to the apical meristem to promote floral initiation^4, 5^. In the apical meristem, it interacts with the FLOWERING LOCUS D (FD) to promote flowering^6^. LEAFY (LFY) is a transcription factor that determines the floral meristem identity and is strongly expressed in the flower buds^7, 8^. *APETALA1* (*AP1*) is involved in the transition from floral induction to flower formation and constituting a hub in the corresponding network of regulatory genes^9, 10^.

We have previously shown that ROS generated by viologen dichloride hydrate (MV)  promotes flowering and increases the expression of *LcAP1* in the floral buds at 18/13 °C (day/night), which is not low enough for the floral induction of litchi^11, 12^, suggesting that ROS treatment could partially bypass the chilling effect, showing potential value for flowering control in the litchi industry. Other than a few ROS responsive EST clones derived from a suppression subtractive hybridization (SSH) library screen^13^, little is known about the transcriptional network controlling litchi flowering underlying ROS.

Previous studies have indicated that both the leaves and shoot apical meristems (SAMs) are involved in the transition from vegetative to reproductive growth. The genetic regulation of these processes in the leaves of annual plants and perennial plants has been widely studied^14^. However, other than studies in Arabidopsis^15, 16^, the global genetic networks at the transcriptome level involved in the transformation from a vegetative meristem (VM) into an inflorescence meristem (IM) in most plant species, including litchi, are still poorly understood.

In this study, potted litchi trees were transferred to growth chambers for low-temperature (LT), medium-temperature (MT), and high-temperature (HT) treatments. Trees at MT were subjected to ROS treatment to promote flowering. RNA-sequencing was applied to obtain a global transcriptome of the apical meristem. The expression profiles of the apical meristems were compared among treatments to identify potential genes involved in the transformation from VM to IM and construct an overview of the gene regulatory network of floral transition potentially co-operated by chilling and ROS.

## Results

### Low temperature induces flowering and MV-generated ROS promotes flowering

As shown in Table 1, after a period of LT treatment, litchi trees were induced to flower with 100% of flowering trees and 95% of flowering terminal shoots, whereas no flowers were observed in the control trees after HT treatment. Under MT conditions, the MV-generated ROS treatment increased the percentage of flowering trees 60%. Moreover, among the flowering trees, the percentage of flowering shoots in ROS-treated trees was much higher than those of the control.

**Table 1:** Effects of chilling and ROS on flowering of litchi trees.

Treatments	Day/night Temperatures	Percentage of flowering trees (%)	Percentage of flowering terminal shoots in the flowering trees (%)
Low temperature (LT)	15 °C/8 °C	100	95.00 ± 1.58
Medium temperature and ROS	18 °C/13 °C	80	94.16 ± 2.22
Medium temperature	18 °C/13 °C	20	10.17
High temperature (HT)	25 °C/20 °C	0	0

### *De Novo* sequence assembly and annotation of a litchi reference transcriptome

Nine RNA-Seq libraries were constructed to identify SAM-expressed genes that are potentially involved in LT-induced and ROS-promoted flowering. As shown in Table 2, we have generated 4.93–5.78 × 10^9^ 150-nt paired-end sequencing data with a Q20 value higher than 97% for each library. To obtain reference sequences, clean reads from all the libraries were *de novo* assembled, and the rRNA were removed for further analysis. The Trinity package assembled 73,117 unigenes with a mean size of 790 bp, an N50 unigene size of 1416 bp, a minimum length of 201 bp, and a maximum length of 13,198 bp (Supplementary Material Table S1).

**Table 2:** Throughput and quality of RNA-seq of the litchi SAMs from four treatments at three time points.

Treatments	No. of basepairs (10^9^)	Total Reads (10^6^)	Clean Reads (10^6^)	Q20 percentage (%)	GC percentage (%)
0D	5.78	3.86	3.83	98.17	46.61
L30D	5.09	3.39	3.37	98.08	45.73
L75D	5.50	3.68	3.64	97.90	47.14
M30D	5.77	3.85	3.82	97.96	46.51
M75D	4.96	3.31	3.28	97.78	47.94
MM30D	4.93	3.28	3.26	97.95	46.09
MM75D	5.92	3.95	3.92	98.23	44.99
H30D	5.38	3.59	3.56	97.89	48.09
H75D	5.42	3.62	3.58	97.69	48.13

Litchi trees were grown in chambers at different temperatures for the following treatments: 15 °C/8 °C (day/night temperature, 12 h day and 12 h night) as low temperature plus water treatment, 18 °C/13 °C as medium temperature plus water treatment, 18 °C/13 °C as medium temperature plus MV treatment, and 25 °C/20 °C as high temperature plus water treatment. The treatments in the first column of the table respectively indicate 0 d (0D), 30 d of low temperature plus water (L30D), 75 d of low temperature plus water (L75D), 30 d of medium temperature plus water (M30D), 75 d of medium temperature plus water (M75D), 30 d of medium temperature plus MV (MM30D), 75 d of medium temperature plus MV (MM75D), 30 d of high temperature plus water (H30D), and 75 d of high temperature plus water (H75D) treatments.

**Figure 1:**
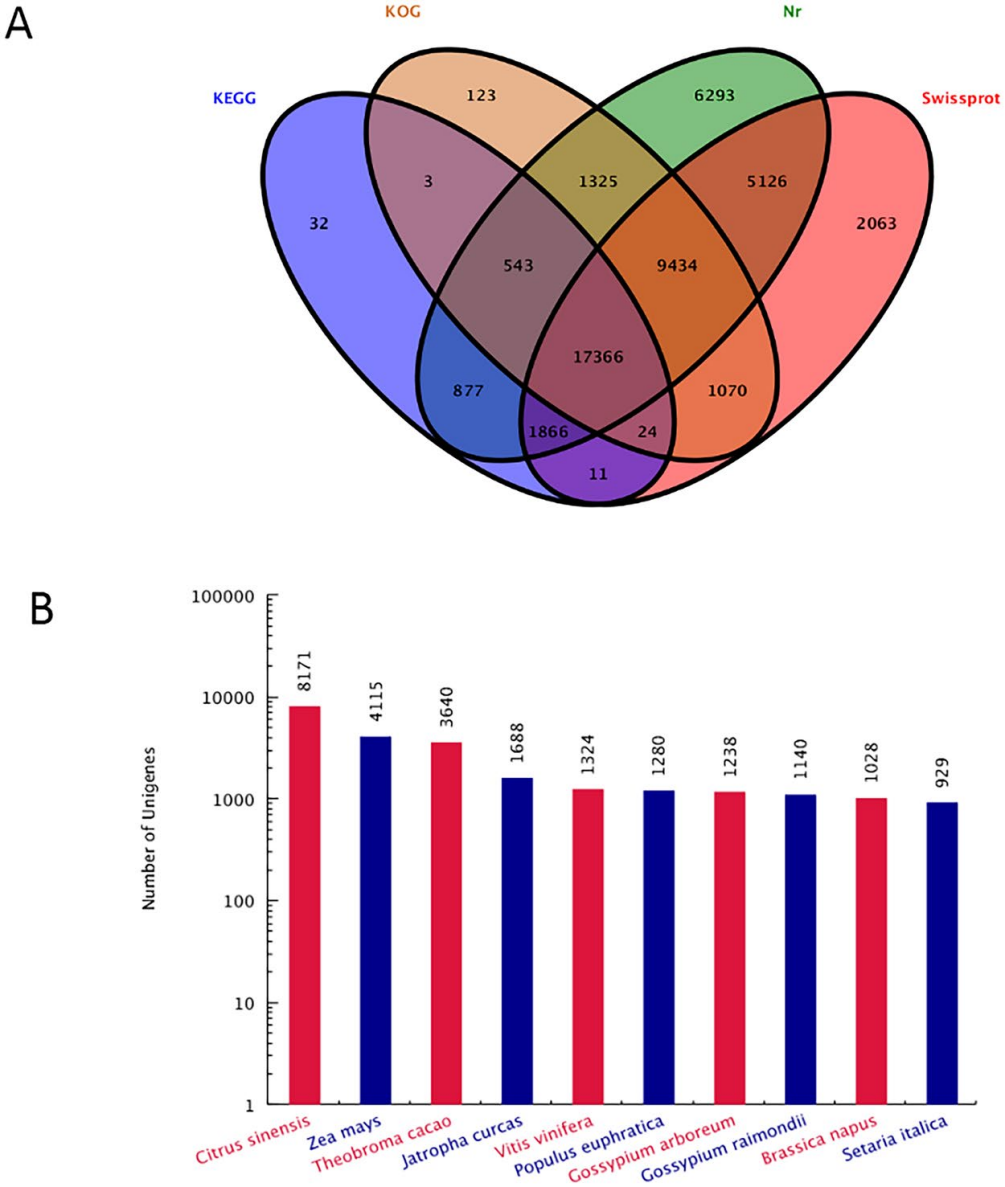
Characteristics of the homology search for litchi unigenes. (**A**) Venn diagram of the number of unigenes annotated using BLASTx with a cut-off E-value 1e^−05^ against protein databases. Numbers in the circles indicate the number of unigenes annotated by single or multiple databases. (**B**) Number of unigenes matching the 10 top species using BLASTx in the nr database.

A total of 42,830 unigenes were annotated by BLASTx using the NCBI nr database, and 36,960 were annotated using the Swiss-Prot protein database. Moreover, 20,722 and 29,888 unigenes were annotated according to the Kyoto Encyclopedia of Genes and Genomes (KEGG) and Cluster of Orthologous Groups of protein (COG) database, respectively. Further, 23.8% (17,366/73,117) of the unigenes were assigned to a homolog in all four databases (Fig. 1A). Based on the NCBI nr database, 25,338 unigenes were annotated to 10 top-hit species. The 4 most top-hit species are *Citrus sinensis*, *Zea may*, *Theobroma cacao*, and *Jatropha curca* (Fig. 1B).

### Identification of differentially expressed genes

**Figure 2:**
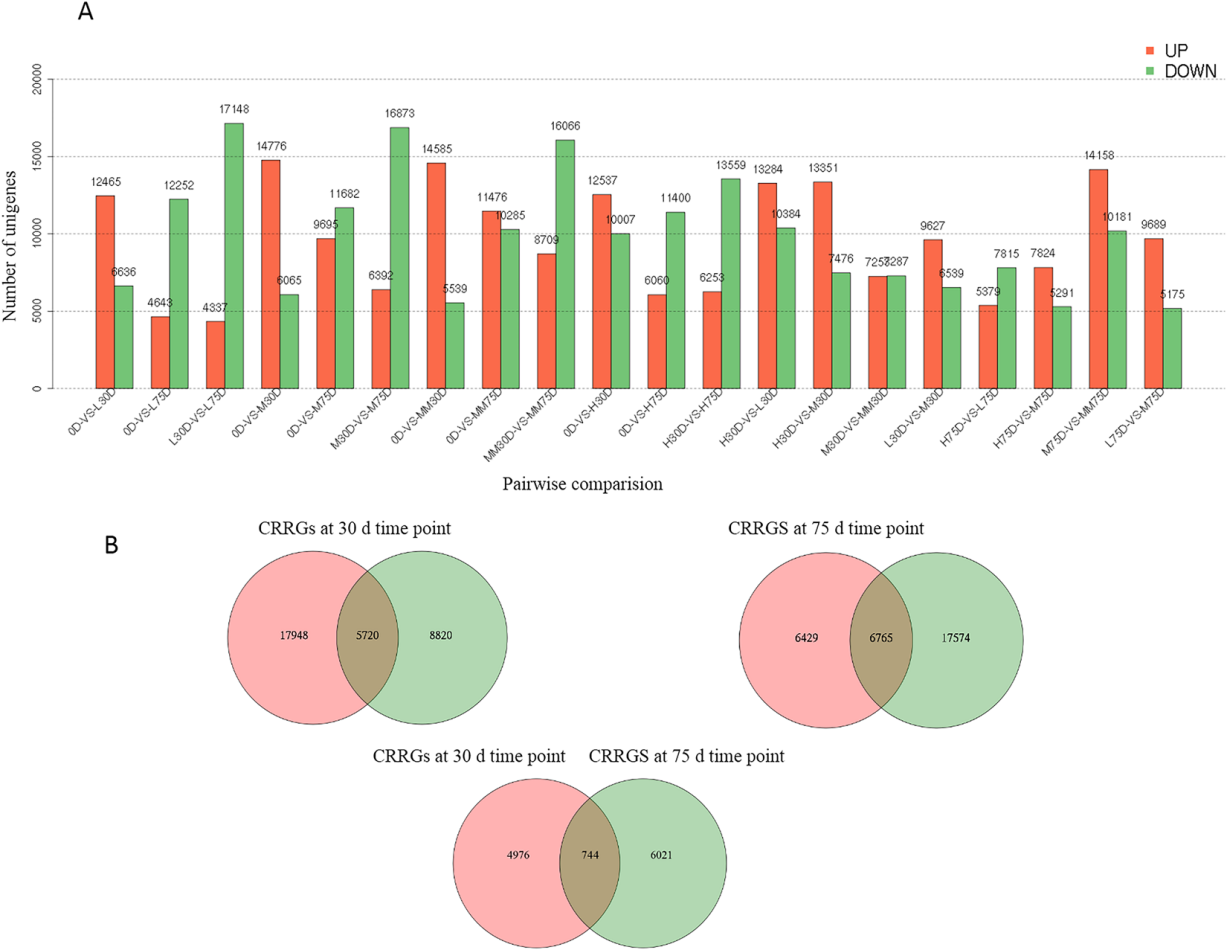
Differentially expressed genes (**A**) and Venn diagram of the differentially expressed genes (DEGs) in responses to low temperature and ROS (**B**). Pairwise comparison of unigene expression was performed using the edgeR package. Significant DEGs were restricted with FDR ≤ 0.05 and the absolute value of log2 Ratio ≥ 1. Among the DEGs, the genes between LT and HT treatments at the same time points were identified as chilling responsive genes (CRGs), and those between ROS treatment at MT and the MT control were identified as ROS responsive genes (RRGs). The overlapping of the CRGs and the RRGs were identified as both chilling and ROS-responsive genes (CRRGs).

Clean reads from the 9 libraries were remapped to the reference sequences. The distribution over different reads abundance categories showed similar patterns among the 9 libraries. More than 58% of the sequences had more than 80% coverage (Supplementary Material Fig. S1). Next, we calculated the unigene expression using the uniquely mapped reads and normalized the results to RPKM. We performed a pairwise comparison, filtered differentially expressed unigenes (DEGs) with FDR ≤ 0.05 and absolute values of log_2_ Ratio ≥ 1. From 0 to 30 d, all samples of the four treatments had more up-regulated unigenes than down-regulated unigenes. However, from 30 to 75 d, the four treatments had more down-regulated unigenes than up-regulated unigenes (Fig. 2A). From 0 to 30 d of treatment, SAMs at LT or at MT plus ROS treatment underwent an early stage of floral induction. From 30 to 75 d, those plants underwent a late stage of floral induction and progressed from floral induction to initiation. The results showed that SAMs at different developmental stages of floral induction had different gene expression patterns.

Among the DEGs, the genes between LT and HT treatments at the same time points were identified as chilling responsive genes (CRGs), and those between ROS treatment at MT and the MT control were identified as ROS responsive genes (RRGs). As shown in Fig. 2B, 23,668 and 14,540 unigenes at 30 d of treatment were found to be responsive to LT and ROS, respectively, with an overlap of 5720 unigenes. Further, 13,194 and 24,339 unigenes at 75 d of treatment were found to be responsive to LT and ROS, respectively, with an overlap of 6,765 unigenes, and 744 unigenes were found to be synchronously responsive to LT and ROS during the all floral induction and initiation stages. Those unigenes overlapping at the 30-d and 75-d time points were pooled together. As a result, 11741 unigenes were identified as both chilling and ROS responsive genes (CRRGs). Among the CRRGs, GO-term analysis indicated that the most top three enriched GO term at the 30- and 75-d time point shared the same subcategories (Supplementary Material Fig. S2). KEGG enrichment analysis showed that the ribosome, oxidative phosphorylation, and unsaturated fatty acids biosynthesis pathways were enriched at 30-d time point, whereas the ribosome, DNA replication, anthocyanin biosynthesis, and sesquiterpenoid biosynthesis pathways were enriched at 75-d time point (Supplementary Material Fig. S3).

### Identification of the flowering related CRRGs

**Figure 3:**
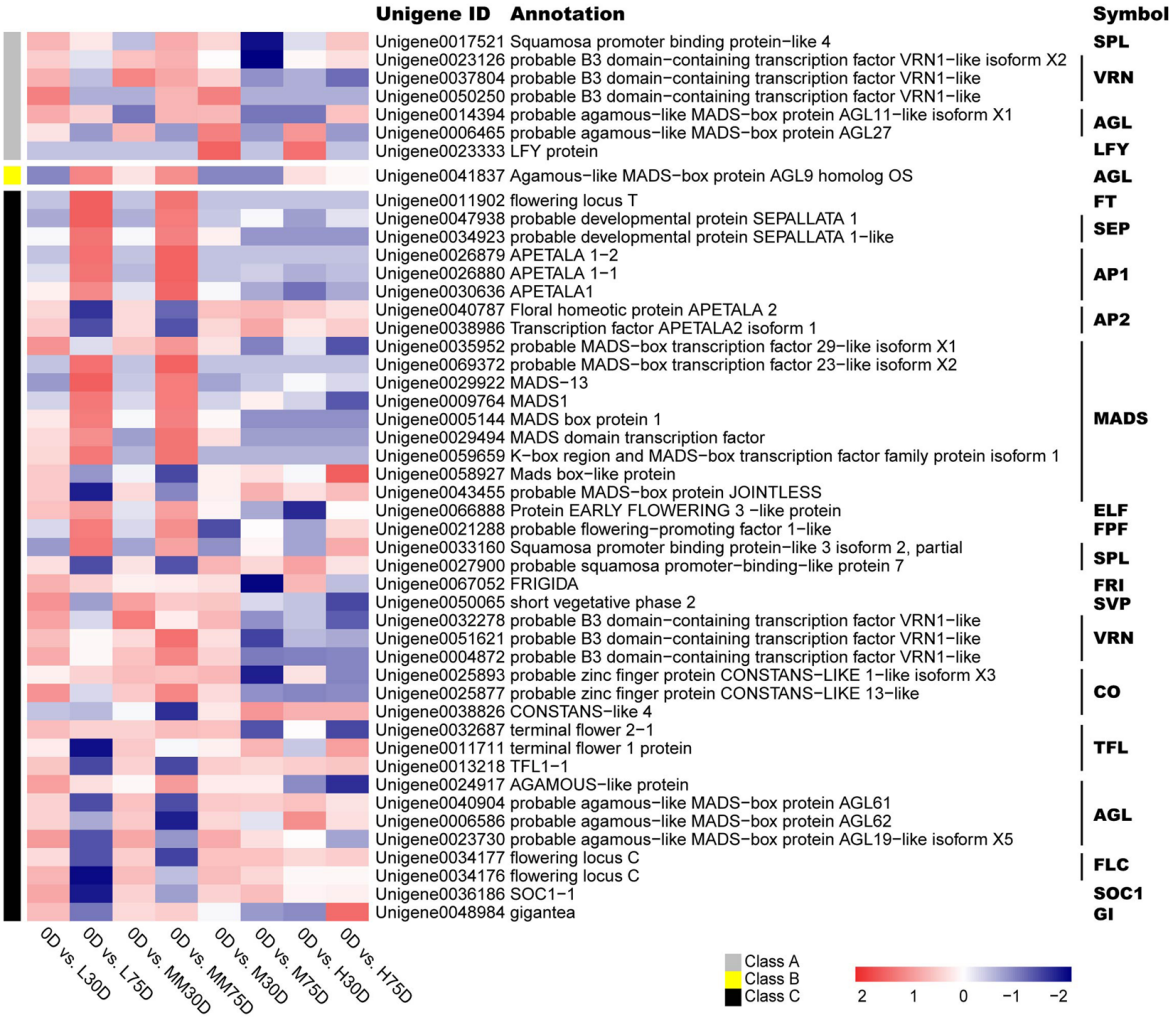
Heat map diagram showing the genes expression profiles of the flowering-related CRRGs. Litchi trees were grown in chambers at different temperatures for the following treatments: 15 °C/8 °C (day/night temperature, 12 h day and 12 h night) as low temperature plus water treatment, 18 °C/13 °C as medium temperature plus water treatment, 18 °C/13 °C as medium temperature plus MV treatment, and 25 °C/20 °C as high temperature plus water treatment. 0D means before treatment, L30D and L75D mean 30 d and 75 d of low temperature plus water treatment, M30D and M75D mean 30 d and 75 d of medium temperature plus water treatment, MM30D and MM75D mean 30 d and 75 d of medium temperature plus MV treatment, H30D and H75D mean 30 d and 75 d of high temperature plus water treatment. Log_2_ ratios of RPKM values of 30- or 75-d time points to 0-d time point were normalized to Z-score.

Flowering-related genes, according to the model plant Arabidopsis were chosen from the 11741 CRRGs to determine the expression patterns in the SAMs of the four treatments. As shown in Fig. 3, a total of 48 flowering related CRRGs were screened. Of those, seven were early-responsive CRRGs, 40 were late-responsive CRRGs, and one was both an early- and late-responsive CRRG. The early-responsive CRRGs encode homologous proteins including 1 Squamosa Promoter Binding Protein-like 4, 3 VRN1-like, 1 AGL11-like, 1 LFY, and 1 AGL27. The late-responsive CRRGs encode homologous proteins, including 1 FT, 2 SEPALLATA (SEP), 3 AP1, 2 APETALA 2 (AP2), 8 annotated MADS-box protein, 1 probable MADS-box protein JOINTLESS, 1 Early Flowering Like protein (ELF), 1 Flowering-Promoting Factor 1-like (FPF), 2 Squamosa Promoter Binding Protein like (SPL), 1 FRIGIDA (FRI), 1 Short Vegetative Phase (SVP), 3 VRN1, 3 CONSTANS-like (CO), 3 Terminal Flower (TFL), 4 AGAMOUS-like (AGL), 2 Flowering Locus C (FLC), 1 SUPPRESSOR OF OVEREXPRESSION OF CONSTANS 1 (SOC1), and 1 GIGANTE (GI). The *AGL9* homolog was observed as synchronously responsive to chilling and ROS during all developmental stages. Overall, there were more late-responsive CRRGs than early-responsive ones. Expression patterns of the genes in the SAMs of LT-induced or the ROS-promoted flowering trees showed different trends compared to those of the HT or MT controls. For example, the expression patterns of the litchi homologs of *FLC* (*LcFLC*, Unigene0034176 and Unigene0034177) and *AP2* (*LcAP2*, Unigene0040787, and Unigene0038986) in the LT-induced or ROS-promoted flowering trees decreased, whereas those in the HT or MT controls showed fewer changes and remained at relatively high levels. The expression patterns of the litchi homologs of *FT* (*LcFT*, Unigene0011902), *SEP* (*LcSEP*, Unigene0047938 and Unigene0034923), and *AP1* (*LcAP1*, Unigene0026879, Unigene0026880, and Unigene0030636) in LT-induced or ROS-promoted flowering trees increased, whereas those in the HT or MT controls showed fewer changes and remained at relatively low levels.

### Identification of the plant hormone signal transduction and biosynthesis-related CRRGs

**Figure 4:**
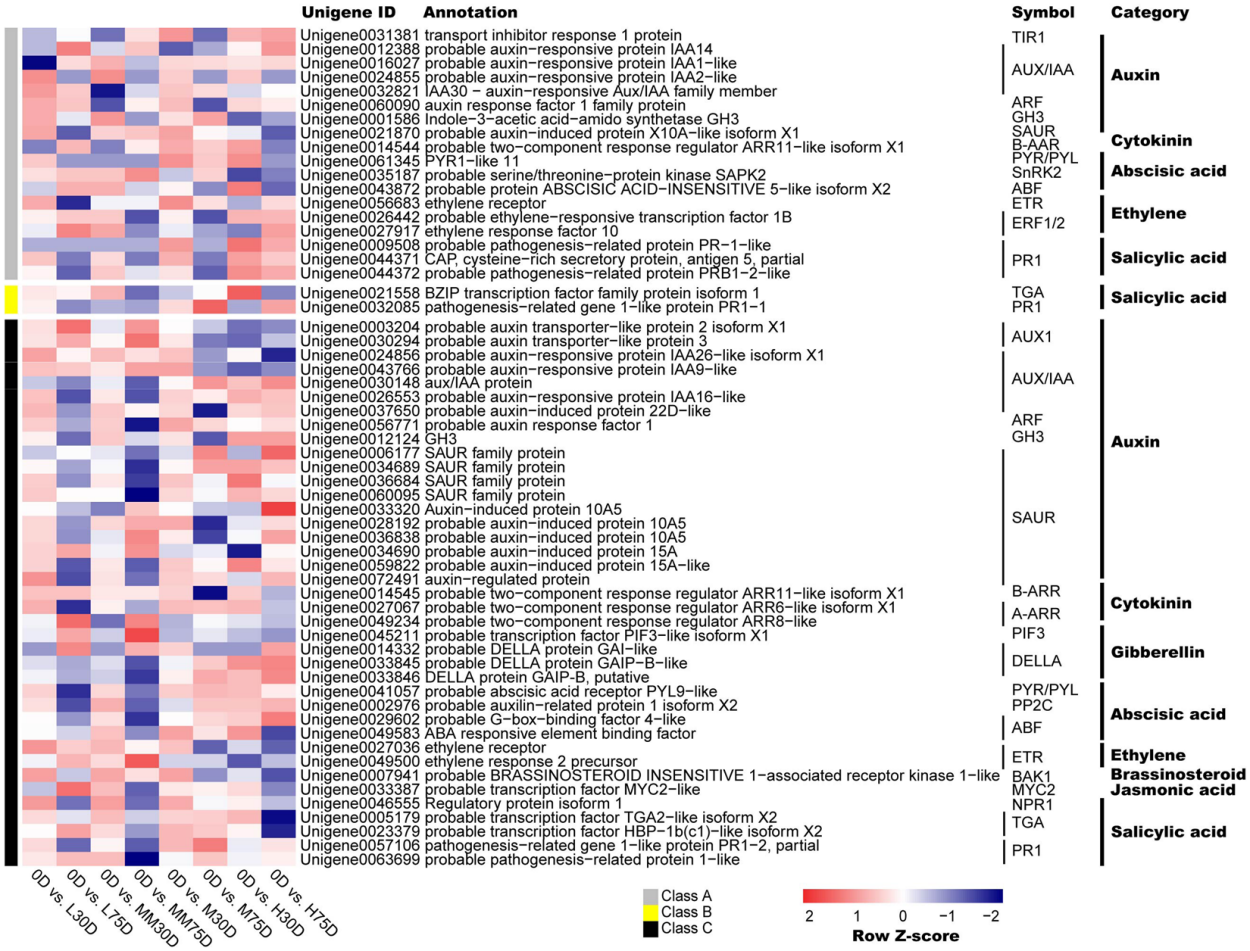
Heat map diagram showing the genes expression profiles of the plant signal transduction-related CRRGs according to the enriched KEEG pathways. Log_2_ ratios of RPKM values of 30- or 75-d time points to 0-d time point were normalized to the Z-score.

Plant hormone signal transduction and biosynthesis-related CRRGs were chosen from the 11741 CRRGs according to the KEGG pathway (Supplementary Material Fig. S4). A total of 59 unigenes related to plant hormone signal transduction were found to be synchronously responsive to chilling and ROS. Of those, 18 were early responsive CRRGs, including 8 auxin (IAA), 1 cytokinine (CTK), 3 abscisic acid (ABA), 3 ethylene, 3 salicylic acid (SA) signal transduction component-encoding genes. A total of 39 unigenes were late-responsive CRRGs, including 19 IAA, 3 CTK, 4 gibberellin acid (GA), 4 ABA, 2 ethylene, 1 brassinosteroid (BR), 1 jasmonic acid (JA), and 5 SA signal transduction component-encoding genes. Two unigens were both early- and late-responsive CRRGs, and these genes encode the SA signal transduction components TGA and PR1, respectively (Fig. 4). There were more late-responsive CRRGs than the early-responsive ones. For example, no GA, BR, and JA signal transduction-related genes were found in the early-responsive CRRGs dataset, but these genes were found in the late-responsive dataset.

Among all the hormone signal transduction-related CRRGs, the IAA genes, including 1 IAA receptor TIR1, 2 AUX1, 9 AUX/IAA, 2 ARF, 2 GH3, and 11 SAUR-encoding genes, were the most abundant, followed by the SA genes, including 6 PR1, 3 TGA, and 1 NPR1-encoding unigenes. The BR and JA genes were the least abundant, as only the *BAK1* and *MYC2* were found. The signal transduction-related CRRGs of CTK included 2 B-AAR and 2 A-AAR encoding genes, while the CRRGs of ABA included 2 ABA receptors PYR/PYL, 1 SnRK2, 1 PP2C, and 3 ABF. Regarding ethylene, the signal transduction-related CRRGs included 3 receptors ETR- and 2 ERF1/2-encoding genes. Most of the signal transduction-related genes in the SAMs of LT-induced or ROS-promoted flowering trees showed different expression patterns, compared to those of HT or MT controls (Fig. 4).

A total of 146 unigenes related to plant hormone biosynthesis were observed as be synchronously responsive to chilling and ROS, including 29 IAA, 9 CTK, 8 GA, 13 ABA, 52 ethylene, 3 BR, 19 JA, 22 SA-related genes. Among these genes, 4 unigenes were related to both IAA and SA biosynthesis, and another 5 unigenes were associated with both ethylene and SA biosynthesis. The biosynthesis-related gene expression showed different trends in the SAMs of the LT-induced or ROS-promoted flowering trees, compared to those of HT or MT controls. Overall, most of the late CRRGs of the IAA, GA, and ethylene biosynthesis-related genes of LT and MV plus MT treatments showed down-regulated expression, whereas those in the HT and MT controls remained at high levels and were relatively stable (Supplementary Material Figs S5–8).

### Confirmation of unigene expression using qRT-PCR

**Figure 5:**
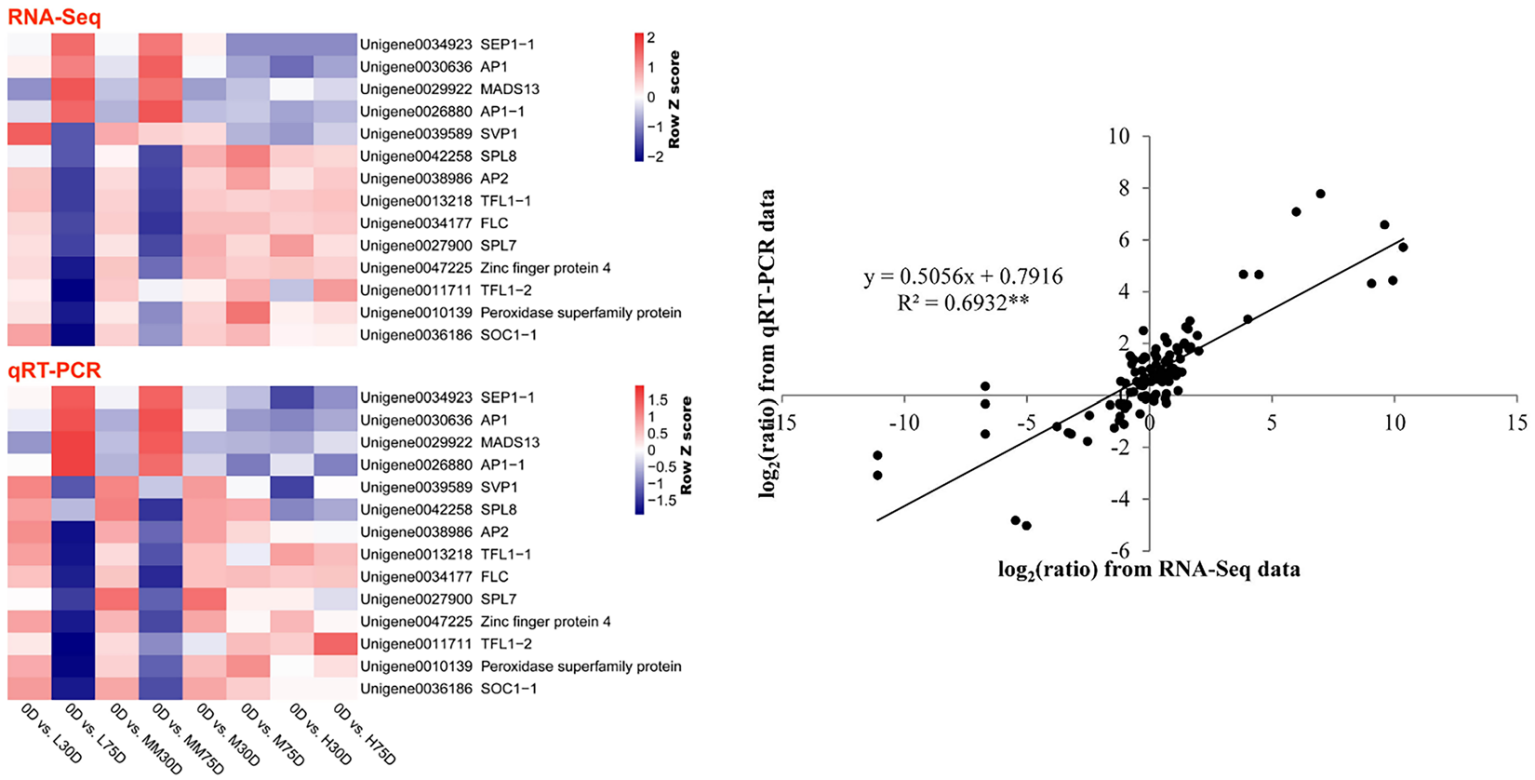
Heat map diagram of the candidate gene expression levels revealed using qRT-PCR and RNA-seq (**Left**), and the correlation between qRT-PCR and RNA-seq of the 14 selected genes ﻿(Right). Fold-changes in the gene expression levels at 30- or 75-d time points compared to that at 0-d time point are indicated by color bars. Scatterplots represent the fold-changes in the gene expression levels at 30- or 75-d time points comparing to that at the 0-d time point.

To validate the accuracy and reproducibility of the transcriptome analysis results, 14 unigenes were selected for qRT-PCR confirmation. As shown in Fig. 5, the expression profiles of the candidate unigenes revealed using qRT-PCR data were consistent with those derived from sequencing. Linear regression analysis of the fold-change in the gene expression ratios between RNA-seq and qRT-PCR showed significantly positive correlation (R^2^ = 0.6932), suggesting a reliable transcriptome analysis using RNA-seq.

### Expression profiles of the candidate genes in early- and late- flowering litchi cultivars

**Figure 6:**
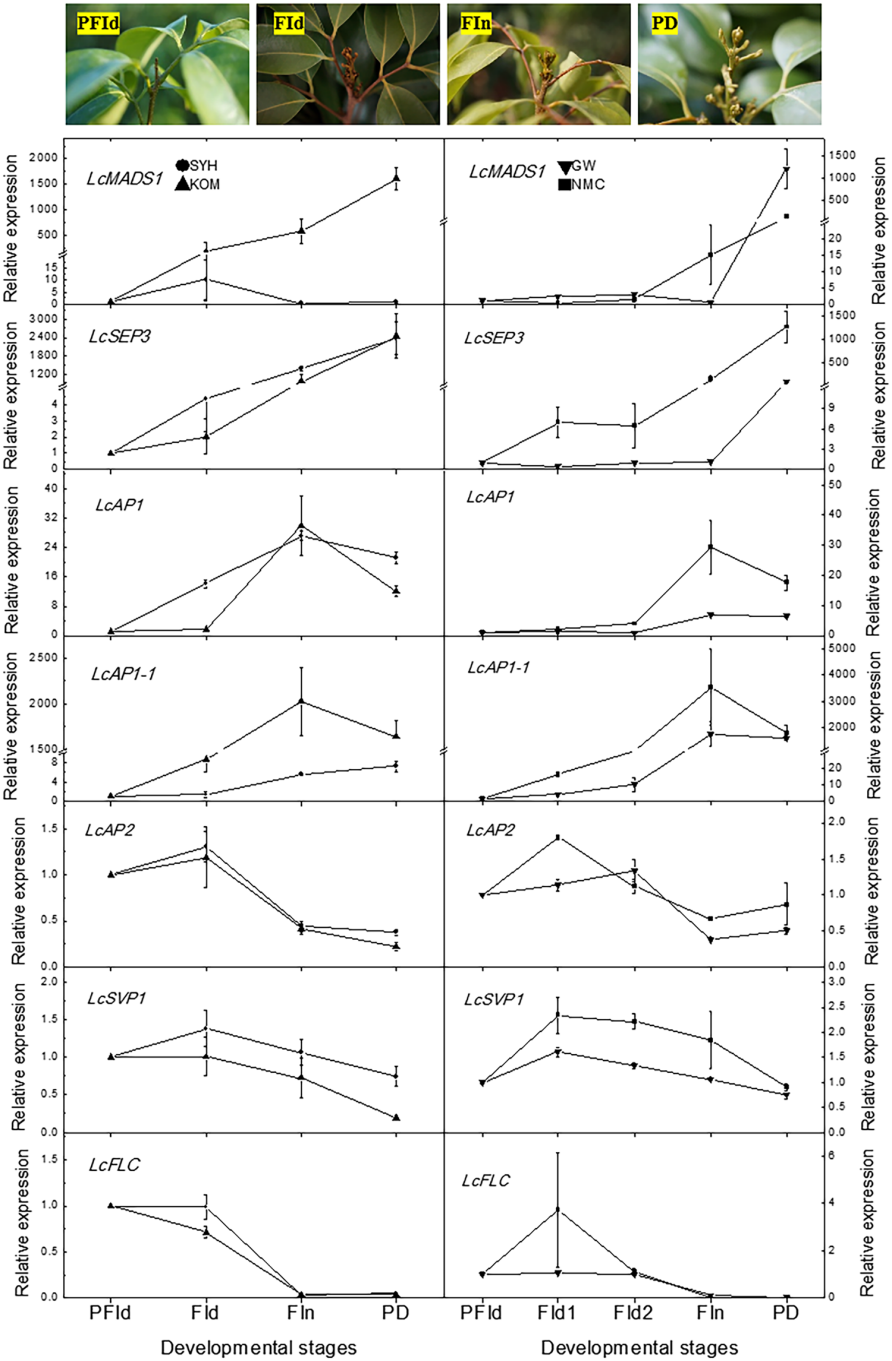
Expression levels of the candidate genes in the SAMs of the early flowering ‘Sanyuehong’ and ‘Kom’, and the late flowering ‘Nuomichi’ and ‘Guiwei’. The SAMs of the early flowering cultivars were collected on September 11 as the PFId stage, October 14 as the FId stage, November 11 as the FIn stage, and December 9 as the panicle developmental (PD) stage in 2015. Those of the late flowering cultivars were collected on September 11 as the PFId stage, November 11 as FId stage 1 (FId1), December 9 in 2015 as FId stage 2 (FId2), February 2 as the FIn stage, and February 21 in 2016 as the panicle developmental (PD) stage. Data are presented as the means of three replicates and the bars represent SE.

**Figure 7:**
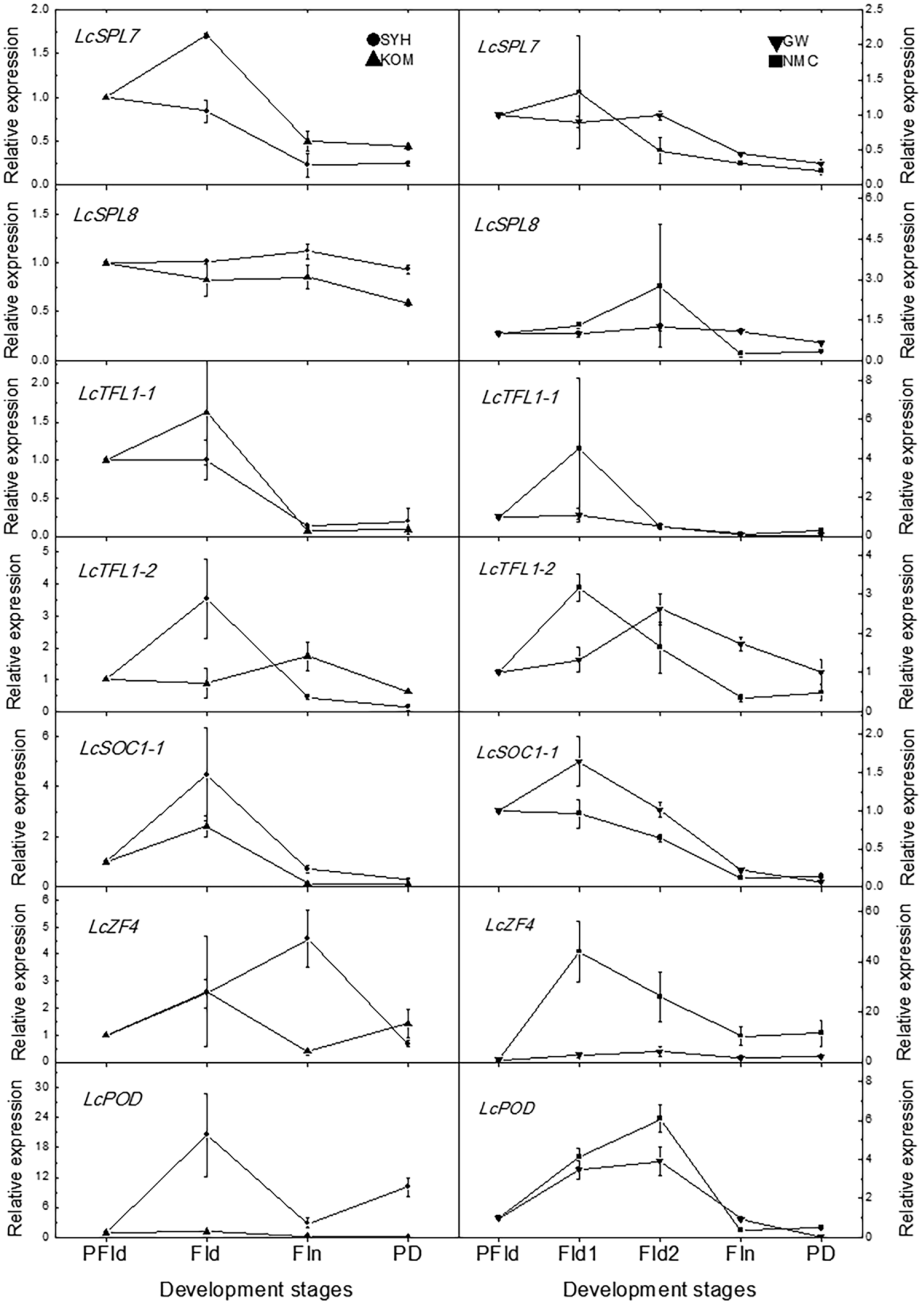
Expression levels of the candidate genes in the SAMs of the early flowering ‘Sanyuehong’ and ‘Kom’, and the late flowering ‘Nuomichi’ and ‘Guiwei’. The SAMs of the early flowering cultivars were collected on September 11 as the PFId stage, October 14 as the FId stage, November 11 as the FIn stage, and December 9 as the panicle developmental (PD) stage in 2015. Those of the late flowering cultivars were collected on September 11 as the PFId stage, November 11 as FId stage 1 (FId1), December 9 in 2015 as FId stage 2 (FId2), February 2 as the FIn stage, and February 21 in 2016 as the panicle developmental (PD) stage. Data are presented as the means of three replicates and bars represent SE.

We subsequently selected candidate genes from the CRRGs data according to their expression patterns. Compared to HT and MT controls, the expression of the unigenes homologious to *MADS1* (*LcMADS1*), *SEPALLATA1-1* (*LcSEP1-1*), *AP1* (*LcAP1*), and *AP1-1* (*LcAP1-1*) in the SAMs of LT- or ROS-treated flowering trees showed increasing trends, while those of the unigenes homologous to *AP2* (*LcAP2*), *SVP1* (*LcSVP1*), *FLC* (*LcFLC*), *SPL7* (*LcSPL7*), *SPL8* (*LcSPL8*), *TFL1-1* (*LcTFL1-1*), *TFL1-2* (*LcTFL1-2*), *SOC1* (*LcSOC1*), *Zinc Finger Protein 4* (*LcZF4*), and *Peroxidase* (*LcPOD*) showed decreasing trends, positively or negatively correlated with flowering (Supplementary Material Table S2). Hence these 14 CRRGs were selected as candidate genes that might be involved in flowering. To further confirm whether the expression of these candidate genes was indeed positively correlated with flowering under field conditions and in different genetic backgrounds within litchi, SAMs at pre-floral induction (PFId), floral induction (FId), floral initiation (FIn), and panicle developmental (PD) stages were collected. The expression of the 14 candidate CRRGs in the SAMs of early- and late-flowering cultivars grown in open fields were determined. As shown in Figs 6 and 7, except for *LcMADS1*, whose expression in the SAMs of ‘Sanyehong’ remained at similar levels, *LcSEP3*, *LcAP1*, *LcAP1-1* in the SAMs of early flowering ‘Sanyehong’ and ‘Kom’ and the late-flowering ‘Nuomichi’ and ‘Guiwei’ litchi trees showed increasing expression trends from PFI to the PD stage, similar to those of the LT and MT plus ROS-treated ‘Nuomichi’ in growth chambers, showing a positive correlation with flowering. The expression of *LcAP2*, *LcSVP1*, *LcFLC*, *LcSPL7*, and *LcTFL1-1* in those decreased during the transition from VM to IM, showing a negative correlation with flowering. However, *LcSOC1*, *LcSPL8*, *LcTFL1-2*, *LcPOD*, and *LcZF4* did not show any regular expression patterns during this period. In total, more than 60% (9/14) of the candidate genes in the SAMs of the early or late flowering cultivars showed similar expression trends, positively or negatively correlated with flowering.

### Co-expression network of CRRGs potentially involved in flowering

**Figure 8:**
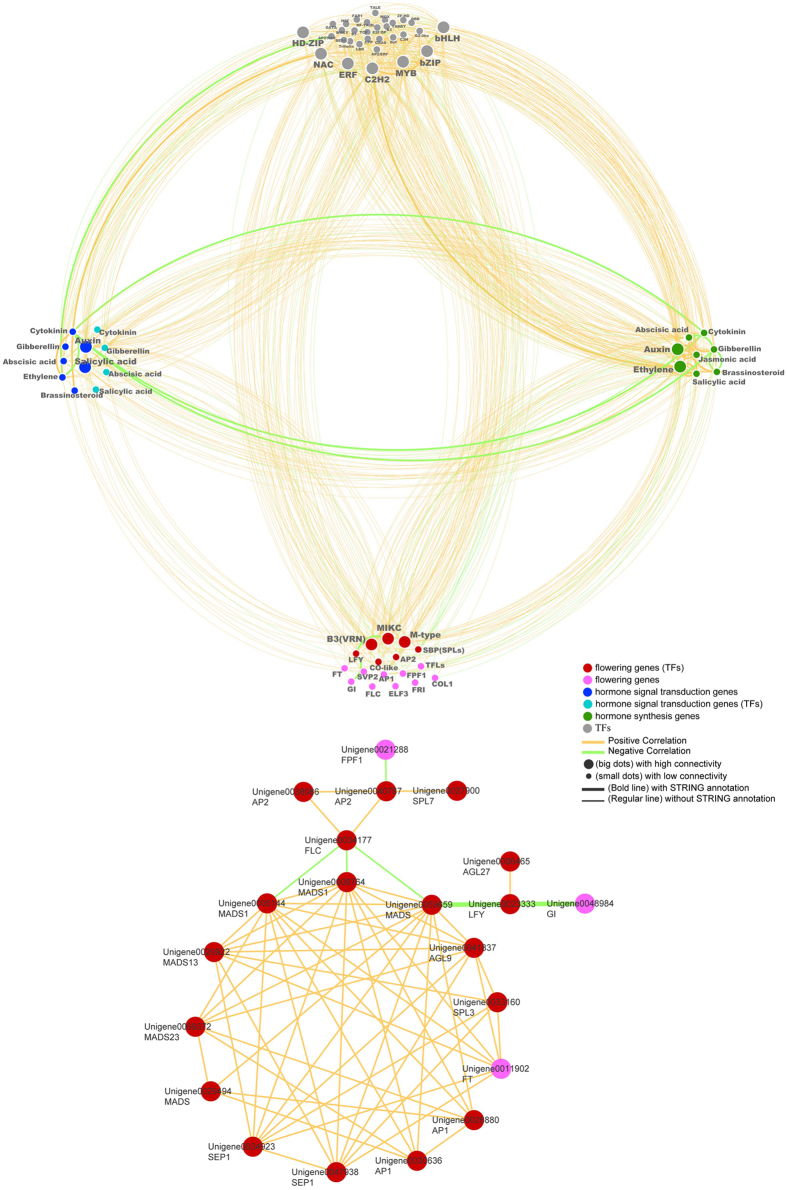
The co-expression network of four groups of genes potentially involved in the chilling-induced and ROS-promoted flowering in litchi. The four groups of genes are TFs (transcription factors), flowering related, plant hormone signal transduction and synthesis-associated genes. The lower image shows that *FLC* is a hub of the flowering-related genes.

Among the 11741 CRRGs, the flowering, the hormone signal transduction and biosynthesis-related genes, and the transcription factors encoding genes which are significantly correlated with each other were pooled together to construct a co-expression network of genes potentially involved in flowering in litchi, including 44, 45, 134, and 207 unigenes uniformly. As shown in Fig. 8, the orange lines and green lines connecting the genes indicate positive and negative co-expression correlations, respectively. The results suggested that these genes are inner-correlated. Moreover, among the flowering-related genes, the litchi *FLC* homolog is a hub, suggesting that LcFLC might be a key flowering regulator in SAMs integratively regulated by ROS and chilling.

## Discussion

At a developmental stage, flowering can be regarded as a transition from vegetative to the reproductive phase. Stress is simply defined as a condition in which the vegetative growth of plants is suppressed. Many of the conditions for floral induction can be considered as stress conditions^17^. Many evergreen woody trees experience environmental stresses such as chilling, drought or oxidative stress, to induce flowering. Water stress induces flowering in mango^18^. Potassium chlorate treatment, which results in oxidative stress, is efficient for flower induction year-around in longan (*Dimocarpus longan*)^19^. The stressed plants do not need to wait for the arrival of a season when photoperiodic conditions are suitable for flowering, and such precocious flowering might be important for species preservation^17^. Hydrogen peroxide is one of the major ROS. It generates as a result of the oxidative stress, derived from environmental stresses, such as excess excitation energy, cold and drought^20^. Hence, it is reasonable that stress-induced flowering may likely be related to ROS. In a previous study, we showed that chilling stress caused H_2_O_2_ accumulation and induced flowering (15/8 °C, day/night), hydrogen peroxide promoted flowering under mildly chilling conditions (18/13 °C, day/night). In the present study, a similar result was found in ROS-treated trees. To identify the integrative chilling and ROS regulatary gene network in the transition from VM to IM, the SAMs from the trees under chilling, medium chilling in the presence or absence of ROS, and non-chilling conditions were collected for RNA extraction and library construction. To overcome the lack of a reference litchi genome, we used all the RNA-Seq data to *de novo* assemble a reference transcriptome. In total, the assembly contained 73,117 unigenes with a mean size of 790 bp. We first identified the chilling responsive genes (CRGs) at the floral induction and initiation stages by screening the differentially expressed genes from the dataset obtained from chilling and non-chilling treated SAMs’. We also identified the ROS responsive genes (RRGs) at those stages by comparing the ROS- and non-ROS-treated SAMs under medium chilling conditions. As a result, we obtained 11741 CRRGs. We identified 48 flowering-related CRRGs, and 205 plant hormone-related CRRGs. Considering that under flowering conditions chilling induced a 100% flowering rate, and ROS promotes a 60% increase in flowering under medium temperature conditions, we checked the expression patterns of the flowering and plant hormone-related genes in the LT, MT plus ROS, MT, and HT SAMs. We selected 14 CRRGs as the candidate genes potentially involved in flowering. Overall, we found that more than 60% (9/14) of the candidate genes in the SAMs of the early or late flowering cultivars showed similar expression trends, positively or negatively correlated with flowering, suggesting that this dataset is applicable for identifying potential genes that are integratively regulated by ROS and chilling underlying flowering.

Among the 48 flowering-related CRRGs, *SEP3* belongs to the E class of MADS-box genes and plays crucial roles in plant development and flowering formation^21^. Thousands of SEP3 binding sites are located in the chromosomes of floral organs, and SEP3 integrates with target MADS-box genes to control flower development^22, 23^. *AP1* is a meristem identity gene whose activity induces the transition of vegetative apices to inflorescence meristems^14^. *AP1* encodes a MADS-domain-containing transcription factor and acts as a hub in controlling the expression of a range of downstream genes to induce floral transitions and flower development in *Arabidopsis* ^9^. AP1 directly represses SVP, SOC1, TFL1^24^, and AP1/SEP3 heterodimers function predominantly, but not exclusively as transcriptional activators during early flower development^9^. In this study, it was found that *LcSEP3* and *LcAP1* in the SAMs of the LT and the MT plus MV-treated trees increased during the VM to IM transition. Consistent with the trees in growth chambers, early flowering ‘Sanyehong’, ‘Kom’ and the late flowering ‘Nuomichi’ and ‘Guiwei’ litchi trees in the open field showed similar expression patterns during the VM to IM transition, suggesting that *LcSEP3* and *LcAP1* might play important roles in the chilling-incuced and ROS-promoted flowering in litchi.

FLC is a MADS-domain-containing transcription factor that acts as a floral repressor^25, 26^. The repression of *FLC* by vernalization involves an initial repression and the maintenance of repression after return to warm temperatures^27^. *VRN1* has been implicated in the initial repression of *FLC* ^28^. *FLC* is expressed at the shoot apex where it represses the expression of SOC1 and FD^29^. It is also expressed to a lesser extent in the leaves where it acts to repress FT^27^. As the *FLC* expression decreases, plants are ready to flower. In our studies, the expression of the litchi *FLC* homolog (*LcFLC*) in the SAMs decreased after LT and MT plus ROS treatments, whereas HT and MT treatments did not decrease the *LcFLC* expression level. Consistent with trees in the growth chambers, early flowering ‘Sanyehong’, ‘Kom’ and the late flowering ‘Nuomichi’ and ‘Guiwei’ litchi trees in the open field showed similar expression patterns, and their *LcFLC* expression level decreased with the seasonal decline in the temperature during autumn and winter, and with the transition from VM to IM. Further, these results showed that *LcFLC* is a hub connecting other flowering-related genes (Fig. 8), suggesting that *LcFLC* as a floral repressor might be involved in the chilling-induced and ROS-promoted flowering. *AP2* might be another floral repressor involved in the transition from VM to IM. In Arabidopsis, AP2-like transcription factors act as repressors of flowering. This clade of proteins comprises AP2 itself, TARGET OF EAT (TOE), and SCHLAFMÜTZE (SMZ) and its paralog SCHNARCHZAPFEN (SNZ)^30–32^. In the LT or MT plus ROS-treated trees, the homolog of *AP2* (unigene0040787 and unigene0038986, *LcAP2*) showed expression patterns similar to those of *LcFLC*. Further, *LcAP2* expression in early- and late-flowering cultivars showed similar profiles, suggesting that the integrative ROS- and chilling regulated *LcAP2* might act as a floral repressor in litchi.

Taking our results and according to the established model in Arabidopsis, most of the flowering-related genes potentially involved in the co-operation of chilling and ROS were found in the simplified flowering time pathways (Supplementary Material Fig. S9) according to the model in Arabidopsis indicated by Blümel *et al*.^33^. LcAP1, LcSPL3, and LcFT might act as floral promoters, whereas *LcFLC*, *LcAP2*, and *LcTFL1* might act as repressors, consistent with the model plant Arabidopsis. However, LcSOC1 showed a different expression pattern, suggesting a different mechanism of flowering might insist in the evergreen woody litchi. Hence, further functional studies should be carried out to investigate the role of the identified genes in litchi flowering.

Plant hormones are signal molecules produced in plant cells in response to environment and are expressed at extremely low concentrations, but regulate a wide range of processes, including determining the formation of flowers, stems, leaves, the development and ripening of fruit, and in response to biotic and abiotic stresses^34^. Plant hormone signals are perceived and transmitted to the nuclear by series signal transduction components to induce gene expression, resulting in a series of physiological processes. In the present study, we identified 205 plant hormone-related CRRGs, including 146 plant hormone biosynthesis regulated and 59 signal transduction component-encoding genes.

Transcription factors are proteins that control the rate of transcription of genetic information from DNA to messenger RNA, by binding to a specific DNA sequence. In turn, this helps regulate the expression of genes near that sequence. These genes may be plant hormone biosynthesis and signal transduction component-encoding genes and flowering-related genes. Interestingly, the gene co-expression network analysis indicated that these genes have inner relationships, suggesting the transition from VM to IM through a regulatory gene network of transcription factors, hormones, and flowering regulators.

In summary, we assembled 73,117 unigenes with a mean size of 790 bp, identifying 11741 unigenes as both chilling and ROS responsive genes (CRRGs). Genes co-expression network analysis indicated that the flowering-related, plant hormone signal transduction and biosynthesis-related CRRGs, and tanscription factors encoding CRRGs have inner relationships.

## Materials and Methods

### Plant material and experiment procedures

Five-year-old air-laying potted trees (*Litchi chinenesis* cv. Nuomici) were grown in 30-L pots with loam, mushroom cinder and coconut chaff (v: v: v, 3:1:1). For sample collection, forty potted trees (1–1.5 m height) were selected for the experiment. When the terminal shoots matured, 10 trees as HT controls were transferred to a growth chamber at 25 °C/20 °C (day/night temperature, 12 h day and 12 h night), a relative humidity of 75–85%, and nature light. Other trees were transferred to growth chambers with the same light and humidity conditions but at 15 °C/8 °C (low temperature, LT), or 18 °C/13 °C (medium temperature, MT). 10 trees at 18 °C/13 °C were sprayed with 60 µM MV (MT plus MV), and 10 trees at the same chamber were sprayed with water as the MT control. The remaining trees at 15 °C/8 °C or 25 °C/20 °C were simultaneously sprayed with water. Trees in 15 °C/8 °C or 18 °C/13 °C chambers were re-warmed to 25 °C/20 °C after 60 d of treatment. At 0 d as the pre-floral induction (PFId), 30 d as the floral induction (FId), and 75 d as floral initiation (FIn) stages, approximately 100 SAMs of the 10 LT- or MT plus MV- treated trees were collected, respectively. At the same time points, similar amounts of SAMs from the LT- or MT-treated trees were collected as controls. All the samples were frozen in liquid nitrogen and stored at −80 °C for RNA extraction.

To determine the effects of the above treatments on scatheless trees, 20 potted trees were subjected to the same treatments. Each treatment had five replicate trees. The percentage of flowering trees and flowering terminal shoots were calculated after treatment.

To study of the candidate gene expression in SAMs of early- and late- flowering litchi cultivars, 14-year-old litchi trees of the early flowering ‘Sanyuehong’ and ‘Kom’, and the late flowering ‘Nuomichi’ and ‘Guiwei’ with similar sizes and phenological stages were selected in the experimental orchard of South China Agricultural University. When the terminal shoots had just matured, the SAMs from three replicate trees of the early flowering cultivars were collected on September 11 as PFId stage, October 14 as the FId stage, November 11 as the FIn stage, and December 9 as the panicle developmental (PD) stage in 2015. The SAMs of the late flowering cultivars were collected on September 11 as the PFId stage, November 11 as FId stage 1 (FId1), December 9 in 2015 as FId stage 2 (FId2), February 2 as the FIn stage, and February 21 in 2016 as the panicle developmental (PD) stage.

### RNA isolation, library construction, and EST sequencing

RNA was extracted using RNA extraction kits from Huayueyang Biotechnology Co., LTD. (China), following the manufacturer’s instructions. The mRNA was enriched using Oligo-dT beads (Qiagen, USA), and fragmented into short fragments by using fragmentation buffer and reverse transcribed into cDNA using random primers. Second-strand cDNA was synthesized using DNA polymerase I, RNase H, dNTPs and buffer. Then, the cDNA fragments were purified using Qiaquick PCR Extraction Kits (Qiagen, USA), end repaired, poly(A) added, and ligated to Illumina sequencing adapters. The ligation products were size selected using agarose gel electrophoresis and fragments ranging from 140 to 200 bp were excised for PCR amplification. The amplified fragments were sequenced using Illumina HiSeqTM 4000 at the Gene Denovo Biotechnology Co. (China).

### De novo assembly and annotation

To obtain clean reads, all raw reads were filtered to remove adapters, and reads with more than 10% of unknown nucleotides were removed and low-quality reads with over 50% low Q-value (≤10) bases were removed. To construct unique consensus sequences as reference sequences, the clean reads from all the samples were *de novo* assembled using the Trinity (Version 2.0) Program^35^. The high-quality clean reads were mapped to ribosome RNA (rRNA) to identify residual rRNA reads. The remaining reads were used for further analysis.

To annotate the unigenes, we used BLASTx program (http://www.ncbi.nlm.nih.gov/BLAST/) at NCBI with an E-value threshold of 1e^−5^ to NCBI nr database, the Swiss-Prot protein database, the KEGG database, and the COG database. The sequence direction of the unigenes was determined according to the best alignment results. When the results conflicted among databases, the direction was determined consecutively using nr, Swiss-Prot, KEGG and COG. When a unigene could not be aligned, the sequence direction was confirmed using the ESTscan program. GO annotation was analyzed using Blast2GO software. Functional classification of the unigenes was performed using WEGO software. KEGG pathway annotation was conducted using BlastX software against the KEGG database. The dataset is available from the NCBI Short Read Archive (SRA) under accession number SRA538256.

### Expression annotation

Clean reads from each sample were uniformly mapped to the reference sequence database using Bowtie2 software. The number of unique-match reads was calculated and normalized to RPKM (reads per kb per million reads) for gene expression analysis. The unigene expression between treatments was compared using the edgeR package (http://www.r-project.org/). Significant DEGs were restricted with FDR ≤ 0.05 and the absolute value of log_2_ Ratio ≥ 1.

### GO and KEGG pathway Enrichment analysis

DEGs were mapped to GO terms in the Gene Ontology database (http://www.geneontology.org/), the gene numbers were calculated for every term, and significantly enriched GO terms in DEGs compared to the genome background were defined using the hypergeometric test.

DEGs were subjected to GO classifications using WEGO^36^, and KEGG^37^ pathway annotation undergone using the Blastall software against the KEGG database.

### Quantitative real-time RT-PCR analysis and gene expression validation

Total RNA was extracted using the Plant Total RNA Isolation Kit (Huayueyang, China). First-strand cDNA was synthesized from 1 μg of extracted total RNA using Reverse Transcriptase M-MLV (RNase H-) (Takara, Japan). Quantitative real-time RT-PCR (qRT-PCR) primers F1/R1 (Table S3) were designed using Primer 6.0 software and synthesized at Sangon Co. Ltd. (Shanghai). The litchi homologue *β-actin* was used as the reference gene (Table S1). The qRT-PCR was performed according to Lu *et al*.^38^ on a CFX real-time PCR machine (Bio-Rad, USA). The qPCR was performed at 95 °C for 3 min, followed by 40 cycles at 95 °C for 15 s and 60 °C for 30 s in 96-well optical reaction plates (Bio-Rad). Each qRT-PCR analysis was performed in triplicate. The transcript quantification of the genes was performed in relation to *Actin* and they were calculated using 2^−△△CT^ method^39^.

For gene expression confirmation, 14 genes were selected for validation by qRT-PCR. Two biological replicates were performed for each gene. Each determination was performed using three technical replicates. A coefficient analysis of fold-change data between qPCR and RNA sequencing was performed, including all the data obtained from the four treatments at the three indicated time points.

### Construction of gene co-expression networks

Pearson’s correlation coefficient between genes was calculated using the R software. A threshold of 0.9 for absolute value of positive or negative correlation coefficients (R) was used as a cutoff to remove low correlations. A gene co-expression network was constructed using the String database (http://string-db.org/) and imaged using Cytoscape (version 3.2.1).

## Electronic supplementary material

Figure S1 to S9 and Table S1 and Table S3

Table S2
