# Supplementary material for: RNA-seq analysis of apical meristem reveals integrative regulatory network of ROS and chilling potentially related to flowering in Litchi chinensis
Source: Sci Rep. 2017 Aug 31;7:10183. doi: 10.1038/s41598-017-10742-y (PMC5579252; doi:10.1038/s41598-017-10742-y)
Supplement: Supplementary file 1 — Figure S1 to S9 and Table S1 and Table S3 [file 41598_2017_10742_MOESM1_ESM.pdf]

Title Page

Title:

RNA-seq analysis of apical meristem reveals integrative regulatory network of ROS and chilling potentially related to flowering in *Litchi chinensis*

The full names of all the authors and their affiliations:

Xingyu Lu[#], Jingjing Li[#], Houbin Chen[1], Jiaqi Hu, Pengxu liu, Biyan Zhou[*]

College of Horticulture, South China Agricultural University, Guangzhou 510642, China

[*]Corresponding author, fax; +86-20-85280228. Email: zhoubiyan@scau.edu.cn

[#]These authors have contributed equally to this work.

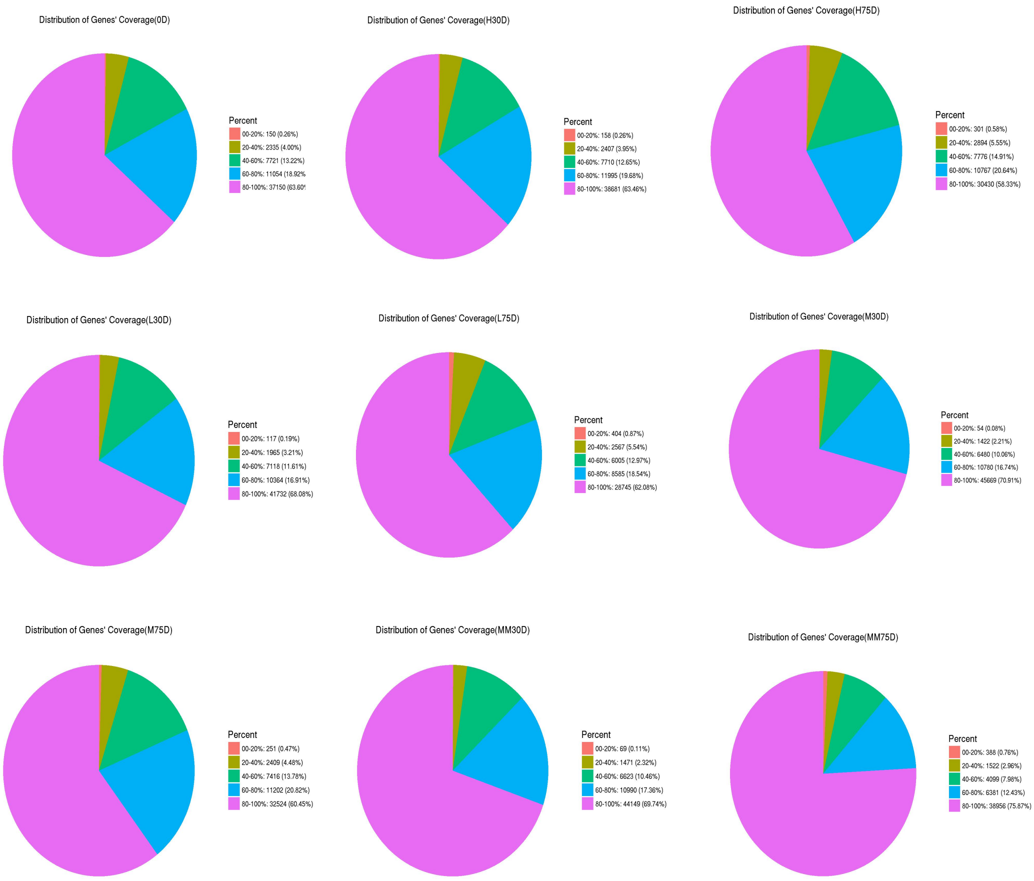

Fig. S1 Distribution of unigenes' coverage in libraries of the 9 samples.

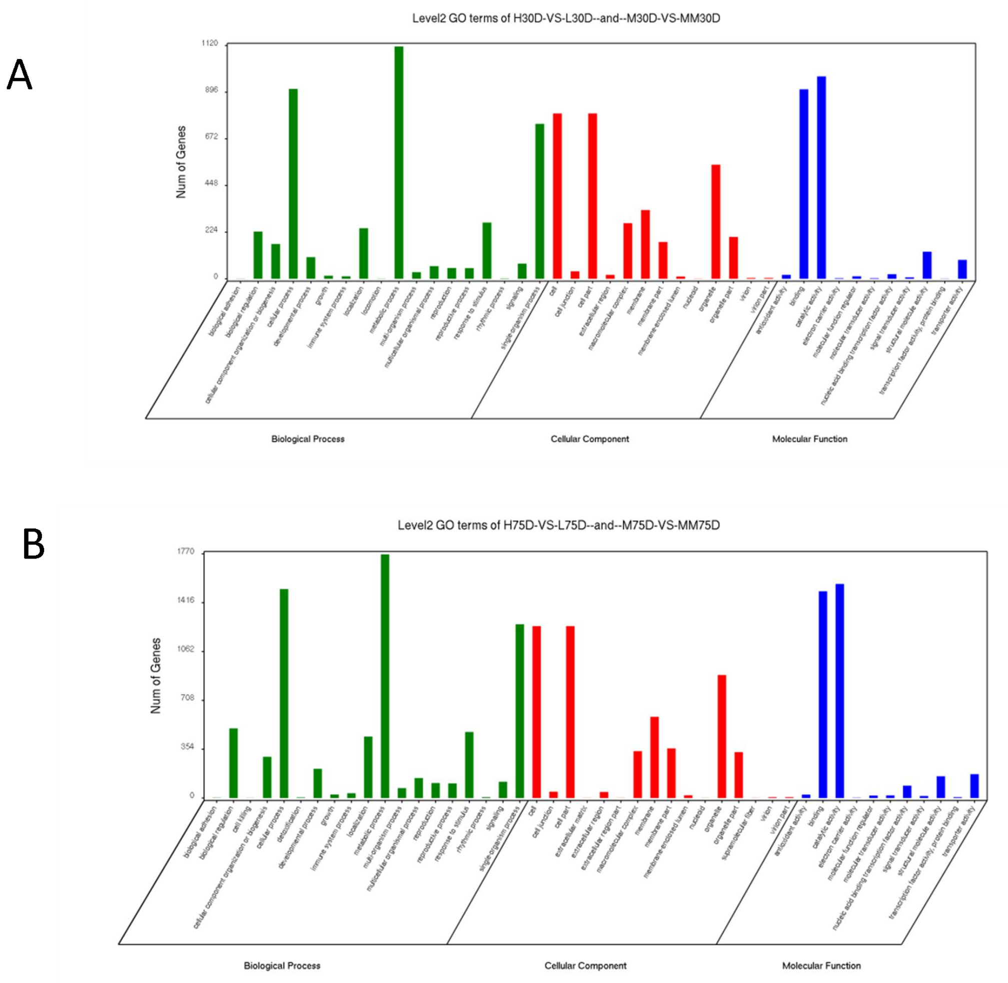

Fig. S2 GO classification of the both chilling and ROS responsive genes (CRRGs) at the 30 d (A) and 75 d (B) time points.

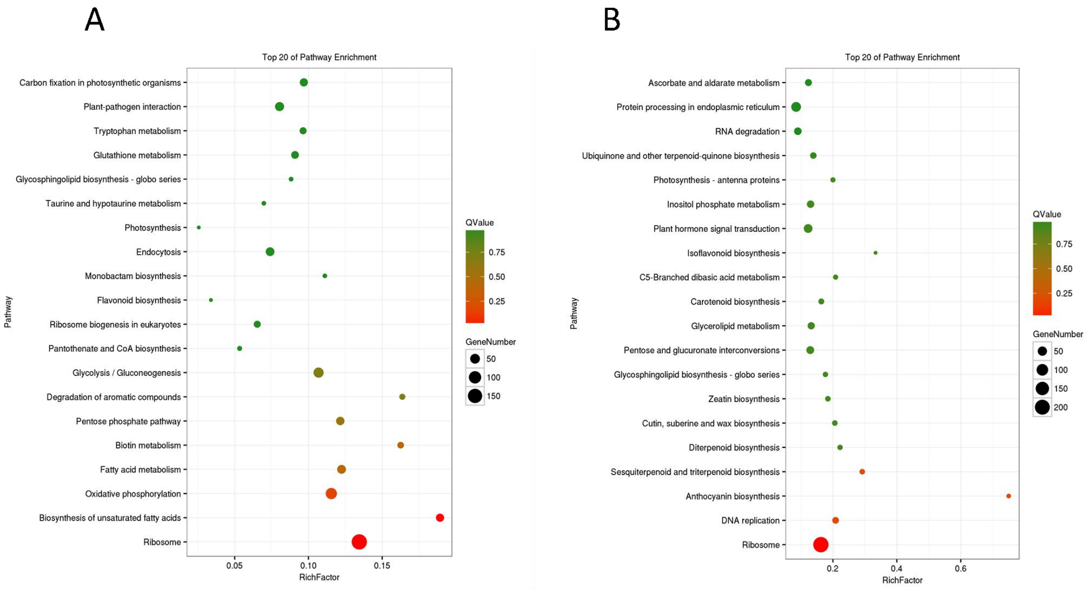

Fig. S3 The 20 top pathway enrichment of the CRRGs at 30 d time point (A) and 75 d time point (B).List of enriched pathways in the at the 30 d (A) and 75 d (B) time points.

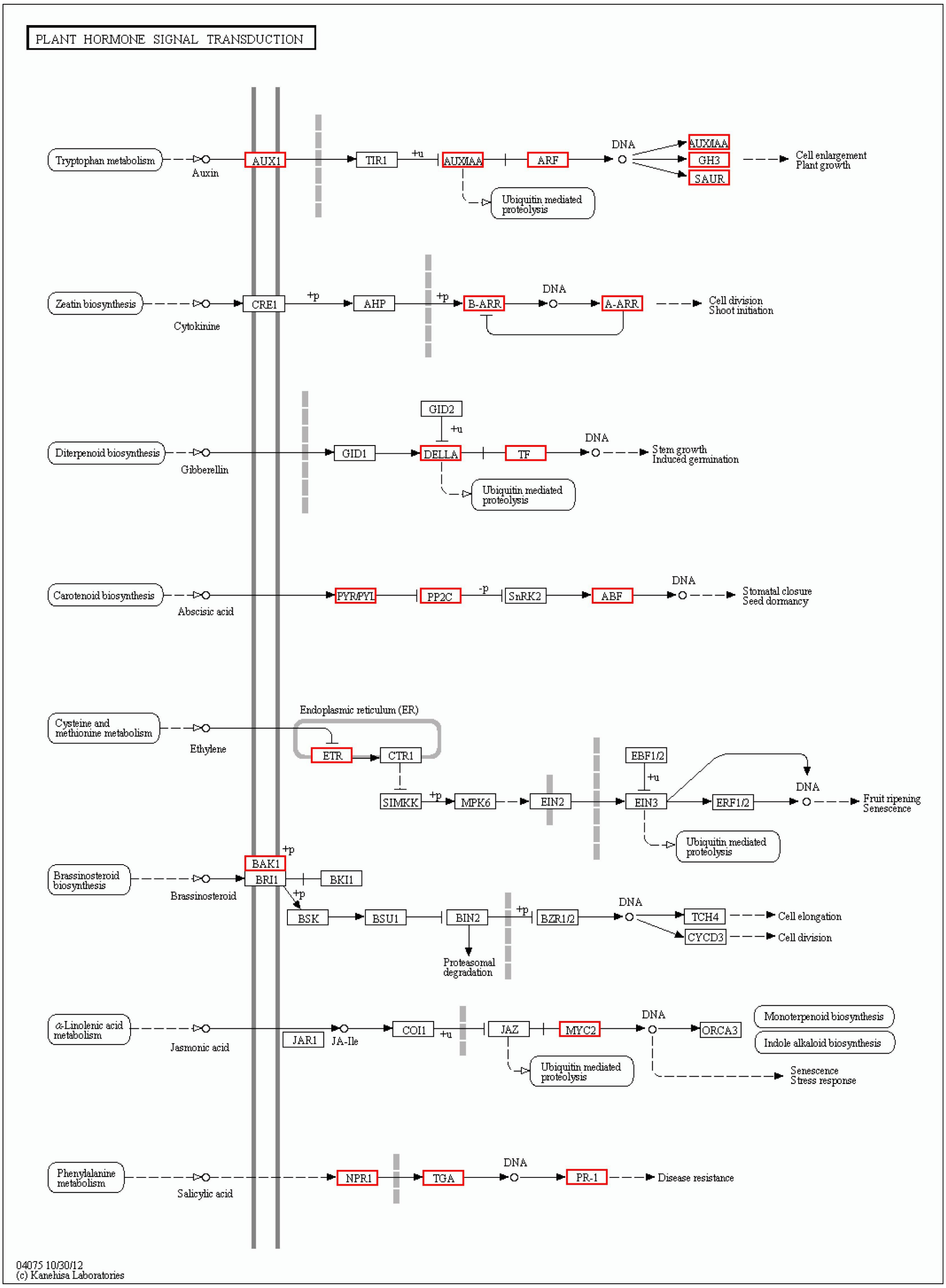

Fig. S4 Plant hormone signal transduction and biosynthesis pathways shown in the KEGG[37] database. The image was obtained from Kanehisa laboratory. The signal transduction components marked with red rectangles are considered to be differentially expressed.

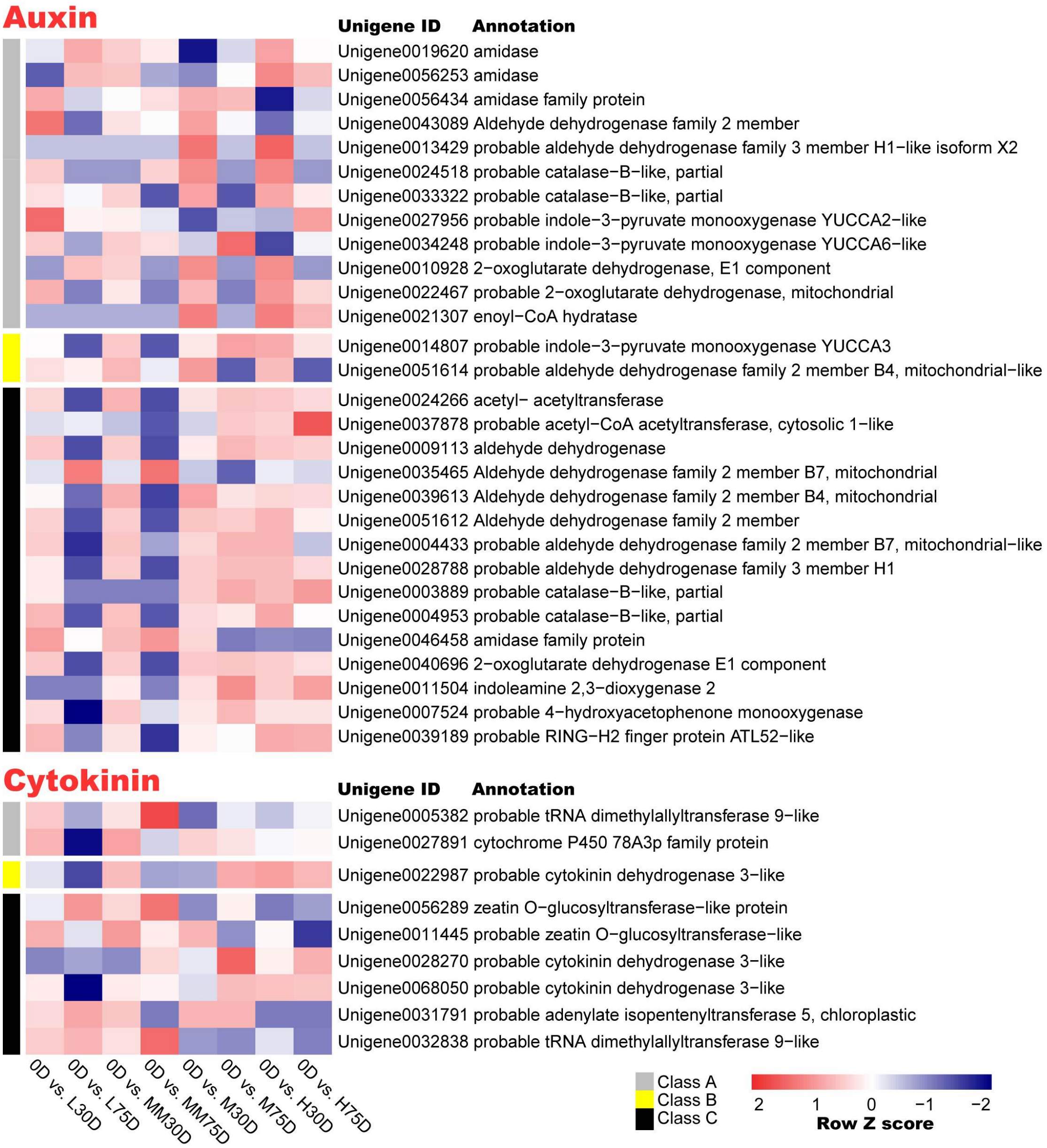

Fig. S5 Heat map diagram showing the genes expression profiles of the auxin and cytokinin biosynthesis related CRRGs. Log2 ratios of RPKM values of 30 or 75 d time points to 0 d time points were normalized to Z-score.

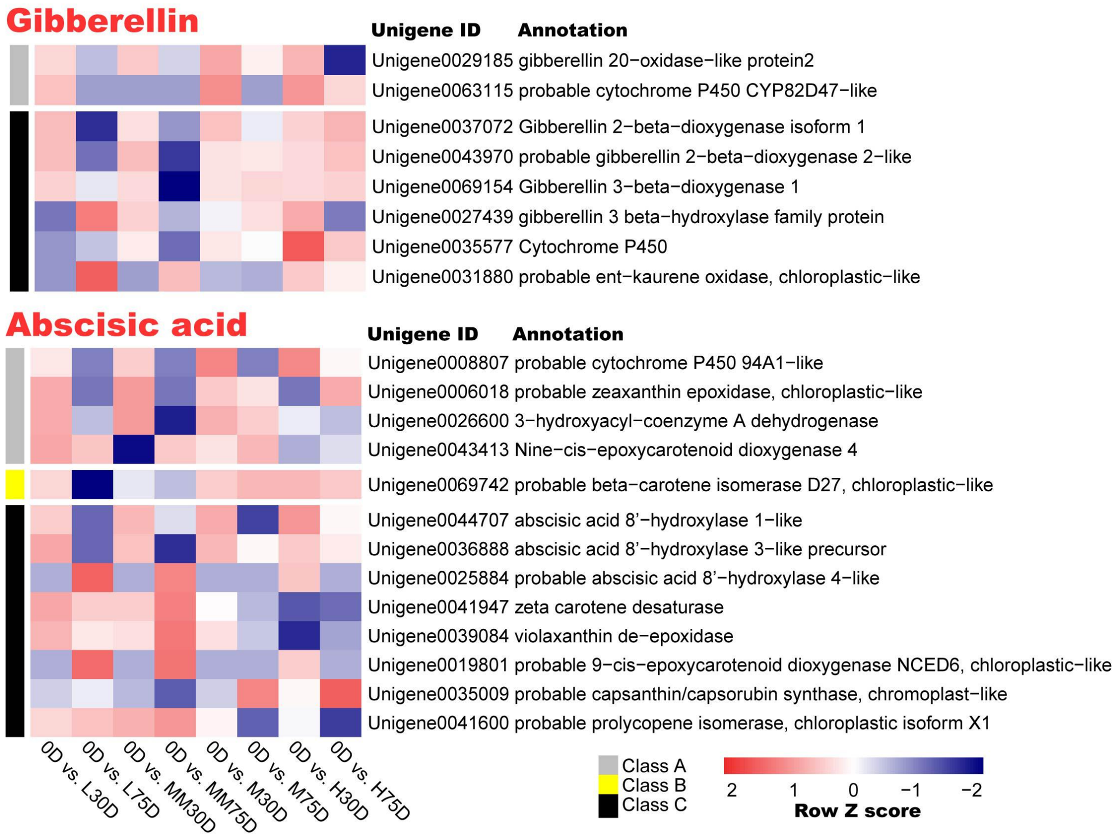

Fig. S6 Heat map diagram showing the genes expression profiles of the gibberellin and abscisic acid biosynthesis related CRRGs. Log2 ratios of RPKM values of 30 or 75 d time points to 0 d time points were normalized to Z-score.

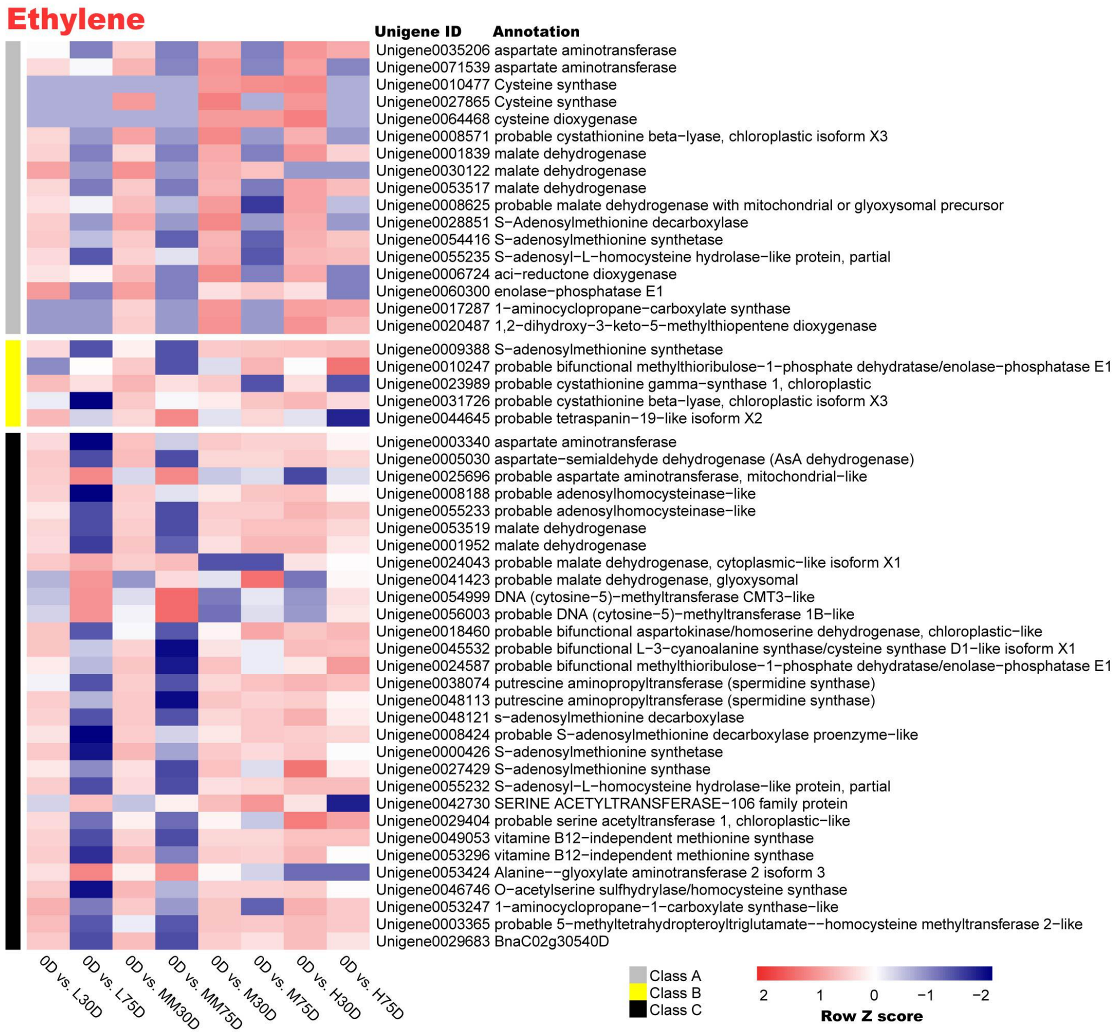

Fig. S7 Heat map diagram showing the genes expression profiles of the ethylene biosynthesis related CRRGs. Log2 ratios of RPKM values of 30 or 75 d time points to 0 d time points were normalized to Z-score.

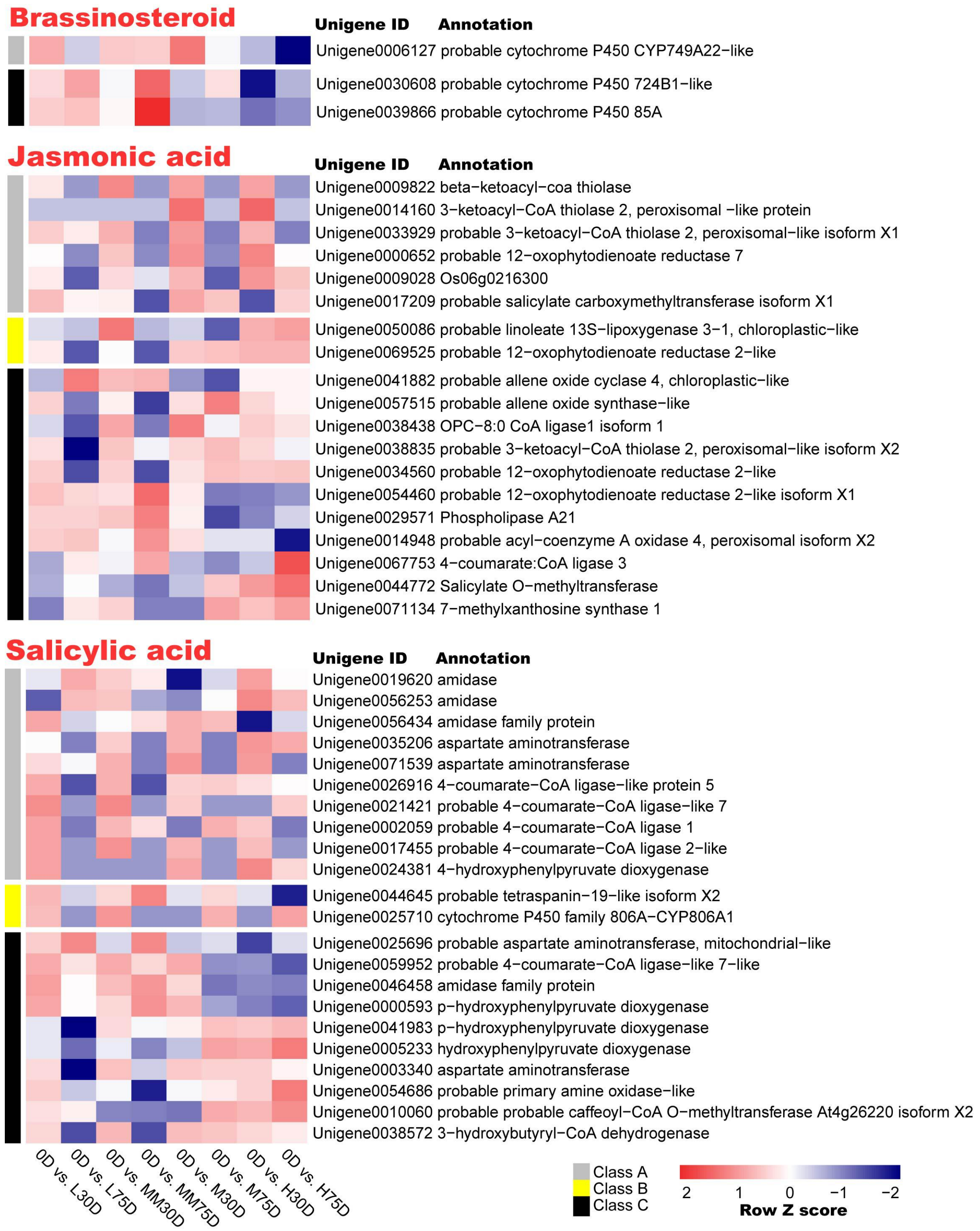

Fig. S8 Heat map diagram showing the genes expression profiles of the brassinosteroid, jasmonic acid, and salicylic acid biosynthesis related CRRGs. Log2 ratios of RPKM values of 30 or 75 d time points to 0 d time points were normalized to Z-score.

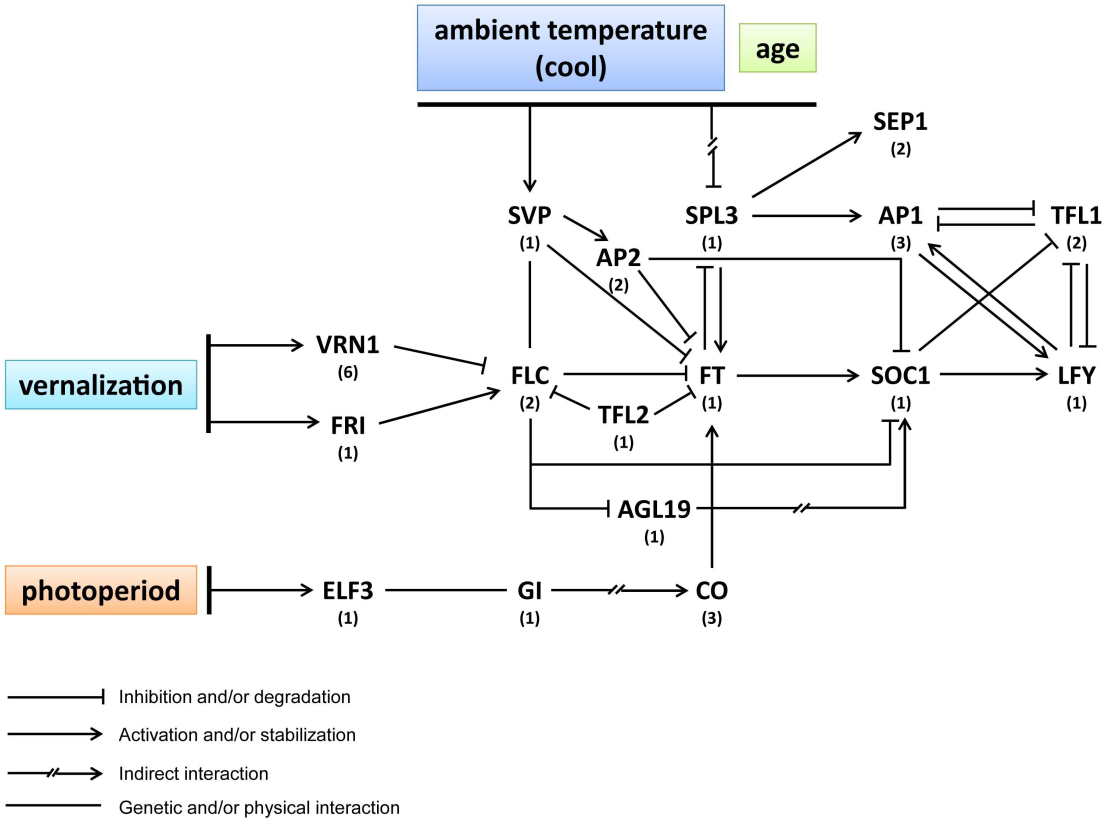

Fig. S9 A simplified diagram showing the four major genetic pathways regulating flowering time in Arabidopsis according to the model indicated by Blümel *et al*[33]. The numbers below each gene represent the members of the identified CRRGs from the RNA-seq dataset. Expression profiles of the detected genes under low temperature, medium temperature, medium temperature plus MV, and high temperature treatments are shown in Fig. 3.

Supplementary Table S1 Summary of the transcriptome assembly

Gene number	N50	Max length	Min length	Mean length
73117	2	13198	201	790

Supplementary Table S3 Primer sequences of the reference gene and candidate unigenes for qRT-PCR

Homology gene	Unigene ID	Sequence F（5´→3´）	Sequence R（5´→3´）
Actin		AGTTTGGTTGATGTGGGAGAC	TGGCTGAACCCGAGATGAT
SEP1-1	Unigene0034923	CGATTGCCAAAATACCCA	GACCAGATAGCAGCCACA
AP1	Unigene0030636	AAAAGAATAGTGGACGAGTT	GAAAGCAAGGGTGGAGGT
MADS13	Unigene0029922	GGAACAACTCCGCAAGAA	TTTGTCCTTCTGTCTCCA
AP1-1	Unigene0026880	CCATTTGTGGGTGTTGTA	GACCTTGCCTTTGTGAGA
SVP1	Unigene0039589	AAAGAAGGTCCGTGTCGT	CAGAGTCGGTTACCAATCA
SPL8	Unigene0042258	CCATAAGGTCTGCGAGTT	TCACGGATGACGACGAAT
AP2	Unigene0038986	TGGGTATGGTGAGACTGAA	TGATGAGGACCTGATTGC
TFL1-1	Unigene0013218	GAAACTGCCGCAAGGAGA	AAGATAGGTAGTGAGGAGGG
FLC	Unigene0034177	TGGAATCAATAGTGACCCTC	CTGCCGGTTGGATGTGCT
SPL7	Unigene0027900	GCCACAATGAGCGTAGAA	ACAAAGTGACGAGCCAGA
Zinc finger protein 4	Unigene0047225	GATACGATGAAGGTTTGG	TTGCTCTGAAGGATACGC
TFL1-2	Unigene0011711	GCAGAAACGAAGGCAGAC	CGTAAACAAGACAGCCAGTA
Peroxidase superfamily protein	Unigene0010139	GCTTTCACCACTTCATCACT	CTAAGGAGCGTAGACAGACC
SOC1-1	Unigene0036186	TTGTCTGATTGATTGACCCTC	TGAGTGTTGGGTGGTGCT
SEP3	Unigene0041837	AGGTTGAGGTGGTTGACG	TCGAAAGGCAGCTTGATA
MADS1	Unigene0005144	GAGTTGGAAGAAACGCATAA	GGCATCTGAGGGTTGTAG
