# Supplementary material for: RNA-seq analysis of apical meristem reveals integrative regulatory network of ROS and chilling potentially related to flowering in Litchi chinensis
Source: Sci Rep. 2017 Aug 31;7:10183. doi: 10.1038/s41598-017-10742-y (PMC5579252; doi:10.1038/s41598-017-10742-y)
Supplement: Supplementary file 2 — Table S2 [file 41598_2017_10742_MOESM2_ESM.pdf]

Title Page

Title:

RNA-seq analysis of apical meristem reveals integrative regulatory network of ROS and chilling potentially related to flowering in Litchi chinensis

The full names of all the authors and their affiliations:
Xingyu Lu#, Jingjing Li#, Houbin Chen1, Jiaqi Hu, Pengxu liu, Biyan Zhou*
College of Horticulture, South China Agricultural University, Guangzhou 510642, China
*Corresponding author, fax; +86-20-85280228. Email: zhoubiyan@scau.edu.cn
#These authors have contributed equally to this work.

Table S2 RPKM values showing the expression levels of the samples.

id	0D_rpkm	L30D_rpkm	L75D_rpkm	M30D_rpkm	M75D_rpkm	MM30D_rpkm	MM75D_rpkm	H30D_rpkm	H75D_rpkm	Description
Unigene000015	0.7418	0.4276	0.1237	1.1088	0	0.5386	0	6.0277	0.4486	PREDICTED: uncharacterized zinc finger protein At4g06634-like [Brachypodium distachyon]
Unigene000037	444.2886	502.7586	73.4838	512.6786	45.9931	336.9058	108.7004	1452.6937	823.7462	Cys2/His2-type zinc finger protein [Citrus trifoliata]
Unigene000042	3.9984	7.1557	0.0794	7.155	5.1306	9.9548	0.7133	7.0467	2.6528	S-adenosylmethionine synthetase [Galdieria sulphuraria]
Unigene000059	2.0735	3.1614	1.0458	2.569	0.3952	1.7809	4.286	0.265	0.1806	p-hydroxyphenylpyruvate dioxygenase [Mangifera indica]
Unigene000065	0.6588	0.0584	0	4.7087	0	0.5214	0	7.2847	0.0547	PREDICTED: 12-oxophytodienoate reductase 7 [Amborella trichopoda]
Unigene000086	6.0725	12.6648	1.9961	11.5089	35.9437	11.0087	2.0866	12.7189	15.73	GmMYB12 family protein [Populus trichocarpa]
Unigene000085	51.6744	79.5256	67.1238	64.3727	42.9068	79.8327	98.302	32.6602	29.8792	WRKY transcription factor 44 [Dimocarpus longan]
Unigene000093	2.1657	0.2497	0	4.1592	0	1.1577	0	1.6545	0	--
Unigene000158	0.5056	1.0409	0.3012	0.5624	1.1098	1.3113	0.1424	0.0818	0.156	Indole-3-acetic acid-amido synthetase GH3.5 [Theobroma cacao]
Unigene000163	22.8446	28.6912	28.7397	23.627	10.3567	26.2176	37.036	14.5424	12.9478	PREDICTED: probable transcription factor KAN2 isoform X2 [Populus euphratica]
Unigene000183	0.6355	0.5233	0	2.4188	0	0.4029	0	5.2401	0.4903	malate dehydrogenase [Nannochloropsis gaditana]
Unigene000195	4.9593	3.2582	0.0556	2.8508	6.0817	5.0017	0.1051	6.2465	2.3326	malate dehydrogenase [Volvox carteri f. nagariensis]
Unigene000205	19.9284	13.2851	25.851	9.4203	4.3266	12.3557	30.3716	3.0345	5.6803	Beta HLH protein 93 [Theobroma cacao]
Unigene000205	0.0751	1.2988	0	0	0.8876	0.6363	0.1481	0.34	0	PREDICTED: 4-coumarate--CoA ligase 1 [Cucumis sativus]
Unigene000206	1.1112	2.514	0	2.0186	2.8235	4.2026	0	3.1598	1.0951	--

Unigene00021	0.425	7.5675	2.9535	3.8239	2.5395	2.2576	1.2105	4.6233	1.4282	PREDICTED: NAC domain-containing protein 100-like [Citrus sinensis]
Unigene00023	6.1224	2.0888	2.8761	2.1267	0.6158	4.4716	2.6151	2.7342	1.2023	PREDICTED: heat stress transcription factor A-7a-like [Gossypium raimondii]
Unigene00024	60.3605	40.4587	16.7381	29.6008	8.0651	44.7358	29.1256	20.9942	88.6505	PREDICTED: ethylene-responsive transcription factor ERF107-like [Citrus sinensis]
Unigene00025	105.7257	90.0783	3.2937	75.5002	4.4666	78.0619	13.5991	211.5751	60.8771	WRKY transcription factor 60 [Gossypium hirsutum]
Unigene00026	4.0037	1.6237	0.1359	22.3511	0	10.0382	0.09	4.9741	0.0845	PREDICTED: similar to Zinc finger protein 271 (Zinc finger protein 7) (HZF7) (Zinc finger protein ZNFphex133) (Epstein-Barr virus-induced zinc finger protein) (ZNF-EB) (CT-ZFP48) (Zinc finger protein [Bathycoccus prasinos]
Unigene00029	0.6291	0.8764	0.082	0.4082	1.2083	1.6815	0.1034	1.2461	1.5289	PREDICTED: auxilin-related protein 1 isoform X2 [Jatropha curcas]
Unigene00032	16.1045	20.3567	41.0912	16.7898	12.6561	14.8572	32.5325	7.834	9.0341	PREDICTED: auxin transporter-like protein 2 isoform X1 [Gossypium raimondii]
Unigene00033	0.6739	2.0637	0	3.6509	2.713	4.6898	0.2076	2.7887	0.9553	aspartate aminotransferase [Sargassum hemiphyllum var. chinense]
Unigene00033	0.1219	2.2955	0	1.603	1.3448	0.0984	0	2.1157	1.4484	PREDICTED: 5-methyltetrahydropteroyltriglutamate--homocysteine methyltransferase 2-like [Musa acuminata subsp. malaccensis]
Unigene00034	40.7502	83.7462	29.146	155.3984	89.5549	115.292	26.456	107.4277	96.4785	PREDICTED: dehydration-responsive element-binding protein 3-like [Citrus sinensis]
Unigene00038	0.7363	0.4598	2.5581	1.242	0.8702	0.5198	0.8468	1.736	1.5904	PREDICTED: zinc finger protein ZAT5-like [Citrus sinensis]

Unigene000388	0	0.118	0	0.3188	1.2098	0	0	0.6372	2.377	PREDICTED: LOW QUALITY PROTEIN: catalase-B-like, partial [Elaeis guineensis]
Unigene000405	3.1635	0.7772	0.7838	0.4307	0.7355	1.7574	1.4827	0.6457	1.9605	PREDICTED: dof zinc finger protein DOF1.2-like [Populus euphratica]
Unigene000438	7.2747	3.3824	1.194	4.3183	4.9164	2.9915	0.5745	4.003	6.034	PREDICTED: protein BEARSKIN1 [Prunus mume]
Unigene000439	0.4891	2.8896	0.0425	2.4328	9.7421	6.3508	0.2712	3.794	0.8804	--
Unigene000441	0.7348	0.2824	0.2979	0	0.1206	0.4447	1.3281	0	0.0441	PREDICTED: B3 domain-containing protein Os01g0234100-like [Prunus mume]
Unigene000442	0.5759	1.8533	0	2.2666	0.9925	2.3232	0.0473	3.5304	1.0107	Zinc finger, GATA-type [Ostreococcus tauri]
Unigene000443	3.0255	6.8223	0.0397	5.6494	12.1216	8.0603	0.3752	11.4788	0.6988	PREDICTED: aldehyde dehydrogenase family 2 member B7, mitochondrial-like [Glycine max]
Unigene000453	0.1432	0.0206	0	0.1487	0.0846	0.4333	0.4589	0.3039	0.4254	PREDICTED: dehydration-responsive element-binding protein 2C [Jatropha curcas]
Unigene000477	0.1773	1.9213	0.037	0.4049	0.2514	1.3733	2.3072	1.7257	0.4596	PREDICTED: dehydration-responsive element-binding protein 2C [Jatropha curcas]
Unigene000487	2.0567	5.182	2.2764	3.3676	0.6265	4.0288	7.8386	0.6479	0.7099	PREDICTED: B3 domain-containing transcription factor VRN1-like [Citrus sinensis]
Unigene000495	0.0568	2.7489	0	0.7367	0.4361	1.9239	0	4.7549	0.2146	PREDICTED: LOW QUALITY PROTEIN: catalase-B-like, partial [Elaeis guineensis]
Unigene000501	0.1025	1.5366	0.2137	0.9047	0.727	2.7919	0	0.6382	0.1107	PREDICTED: similar to Zinc finger protein 271 (Zinc finger protein 7) (HZF7) (Zinc finger protein ZNFphex133) (Epstein-Barr virus-induced zinc finger protein) (ZNF-EB) (CT-ZFP48) (Zinc finger protein [Bathycoccus prasinos]

Unigene000503	0.8096	0.9333	0	0.6303	0.6379	1.4697	0	0.9926	0.5829	aspartate-semialdehyde dehydrogenase (AsA dehydrogenase) [Chondrus crispus]
Unigene000514	0	0.1689	22.257	0.076	0	0.0443	18.812	0	0	MADS box protein 1 [Litchi chinensis]
Unigene000517	0.1646	0.4744	0.2573	0.5126	0.5349	0.6474	0.2231	0.652	0.0444	PREDICTED: transcription factor TGA2-like isoform X2 [Gossypium raimondii]
Unigene000523	6.1161	1.0847	0.3065	0.7326	4.2397	1.1388	0.4058	3.6608	6.5423	hydroxyphenylpyruvate dioxygenase [Coptis japonica var. dissecta]
Unigene000527	19.9882	32.3632	19.3101	28.3829	10.9378	34.0543	36.4547	12.5067	6.8628	Basic helix-loop-helix DNA-binding superfamily protein isoform 3 [Theobroma cacao]
Unigene000538	0.8424	1.4567	0.2509	0.1249	0.5689	1.0195	9.0165	0.3405	0.5849	PREDICTED: tRNA dimethylallyltransferase 9-like [Citrus sinensis]
Unigene000572	1.2168	1.7265	1.0244	2.2348	0.9956	0.2832	1.7532	0.1589	0.2022	PREDICTED: GATA transcription factor 15-like [Gossypium raimondii]
Unigene000576	1.3641	1.8084	2.2038	0.2478	2.257	0.7429	1.9835	0.5403	1.1418	WUSCHEL protein [Dimocarpus longan]
Unigene000586	0.3078	7.4526	8.7438	8.6282	7.6403	4.9365	3.5659	5.9229	2.5768	PREDICTED: scarecrow-like protein 15 [Cucumis melo]
Unigene000589	0.8957	3.6232	0	3.5331	2.1363	6.6418	0.0321	4.2759	0.7387	--
Unigene00060[illegible]	0	0.9572	0	0.3315	0.151	1.7779	0	0	0.9658	PREDICTED: zeaxanthin epoxidase, chloroplastic-like [Pyrus x bretschneideri]
Unigene000612	0.368	1.1878	0.3836	1.8718	0.5219	0.8907	0.8707	0.3332	0.0795	PREDICTED: cytochrome P450 CYP749A22-like [Malus domestica]
Unigene000617	3.5811	3.6697	4.9769	4.1305	11.9916	4.4946	2.2228	3.3025	15.3997	SAUR family protein [Theobroma cacao]
Unigene000635	1.3059	3.0769	2.9133	2.5328	1.1778	2.5647	5.4882	0.9075	1.064	PREDICTED: transcription factor MYB98-like [Prunus mume]
Unigene00064[illegible]	27.6716	9.7038	0.7629	10.6586	0.5286	17.2002	5.4319	26.8082	26.7031	PREDICTED: ethylene-responsive transcription factor ERF017 [Populus euphratica]
Unigene000646	0.9961	0.0741	0	3.3356	0	0.3889	0	1.3456	0	PREDICTED: agamous-like MADS-box protein AGL27 [Prunus mume]

Unigene00065[illegible]	7.3024	5.01	8.6141	7.6914	17.1345	6.8116	2.3528	21.2868	22.507	PREDICTED: ethylene-responsive transcription factor ERF035-like [Citrus sinensis]
Unigene00065[illegible]	1.8466	5.0318	1.2832	4.7633	2.2486	5.5534	0.331	11.2099	4.3516	PREDICTED: agamous-like MADS-box protein AGL62 [Populus euphratica]
Unigene00067[illegible]	2.2794	0.2285	0.1033	8.4361	0	1.3194	0	2.4677	0	aci-reductone dioxygenase [Volvox carteri f. nagariensis]
Unigene00068[illegible]	0.0997	0.115	0	3.2615	0	0.5432	0	1.5804	0	--
Unigene00072[illegible]	2.9396	1.3954	0.0901	0.0898	0	0.6278	3.6651	0.0979	0.5603	bHLH transcription factor [Gossypium hirsutum]
Unigene00073[illegible]	1.1167	0.6897	0	4.2437	0	1.3274	0.0393	1.4219	0	--
Unigene00074[illegible]	1.1303	5.9055	0.0251	6.7657	7.447	7.9752	0.3319	13.0925	2.6495	--
Unigene00075[illegible]	0.3886	0.9919	0	0.6915	2.4274	1.6124	0.1642	0.7853	0.7494	MULTISPECIES: 4-hydroxyacetophenone monooxygenase [Gordonia]
Unigene00077[illegible]	0.8349	1.6041	0	1.04	0.6578	0.8083	1.9205	0.7559	2.5248	BR enhanced expression 1 [Theobroma cacao]
Unigene00079[illegible]	5.5776	12.4817	3.9328	12.0057	2.7143	11.813	7.5201	4.7345	1.7718	PREDICTED: BRASSINOSTEROID INSENSITIVE 1-associated receptor kinase 1-like [Citrus sinensis]
Unigene00080[illegible]	0.9705	0.7459	0	3.7947	1.8352	6.1663	0	1.6659	0.6114	c2h2-type zinc finger-containing protein [Nannochloropsis gaditana]
Unigene00081[illegible]	1.5751	3.038	0	1.6036	4.3317	3.336	0.3583	5.1423	0.916	PREDICTED: LOW QUALITY PROTEIN: adenosylhomocysteinase-like [Beta vulgaris subsp. vulgaris]
Unigene00082[illegible]	4.3654	1.9459	0.455	0.9365	2.1669	0.634	0.8607	2.141	2.326	PREDICTED: transcription factor MYB108-like [Citrus sinensis]
Unigene00084[illegible]	0.4723	0.6125	0	0.5515	1.2558	1.1431	0.1164	1.069	0.8926	PREDICTED: S-adenosylmethionine decarboxylase proenzyme-like [Oryza brachyantha]
Unigene00084[illegible]	21.1854	32.6534	9.3573	31.3113	31.1434	27.5834	8.6403	28.116	20.5831	PREDICTED: transcription factor bHLH63-like isoform X2 [Citrus sinensis]
Unigene00085[illegible]	0.5779	0.1777	0	4.1195	0	1.4921	0	0.9375	0	PREDICTED: cystathionine beta-lyase, chloroplastic isoform X3 [Nicotiana tomentosiformis]

Unigene00086[illegible]	4.8217	1.2762	0.2564	13.5858	0	4.2278	0.0485	9.8562	0.0531	probable malate dehydrogenase with mitochondrial or glyoxysomal precursor [Thalassiosira pseudonana CCMP1335]
Unigene00088[illegible]	0.8523	0.134	0	5.3893	0	0.3282	0	4.7576	0.0628	PREDICTED: cytochrome P450 94A1-like [Solanum tuberosum]
Unigene00089[illegible]	4.4893	10.6131	13.8748	5.7828	7.3405	7.1897	16.0055	4.3643	6.301	PREDICTED: transcription factor bHLH67 isoform X1 [Vitis vinifera]
Unigene00090[illegible]	1.1157	0.2144	0	1.1823	0	0.3938	0.0458	4.3142	0.8284	Os06g0216300 [Oryza sativa Japonica Group]
Unigene00090[illegible]	22.0678	15.4483	1.902	10.1696	1.1322	18.8818	6.4648	28.8355	13.414	Zinc finger family protein [Theobroma cacao]
Unigene00091	0.1918	1.4373	0	0.3982	2.4936	1.1607	0	1.3025	1.0358	aldehyde dehydrogenase [Thalassiosira pseudonana CCMP1335]
Unigene00093[illegible]	9.9938	22.7824	9.6651	12.4805	5.1686	14.3323	36.2832	6.1572	2.8405	Zinc finger family protein [Theobroma cacao]
Unigene00093[illegible]	0	0.7596	0	1.3977	1.4897	0.312	0	1.686	2.135	S-adenosylmethionine synthetase [Chondrus crispus]
Unigene00095[illegible]	0.827	0	0	0.8049	0	0	0	3.5101	0.3908	PREDICTED: pathogenesis-related protein PR-1-like [Camelina sativa]
Unigene00097[illegible]	0.0628	0.0724	66.647	0.0652	0.5199	0.0761	59.2865	0.0711	0	MADS1 [Mangifera indica]
Unigene00098[illegible]	0.68	0.0653	0	1.3531	0	2.9492	0	1.0262	0	beta-ketoacyl-coa thiolase [Phaeodactylum tricornutum CCAP 1055/1]
Unigene00100[illegible]	18.6415	7.7366	6.8397	2.7866	11.2802	3.0683	2.9404	10.296	13.6913	PREDICTED: probable caffeoyl-CoA O-methyltransferase At4g26220 isoform X2 [Gossypium raimondii]
Unigene00102[illegible]	1.4541	0.1578	0.4279	0.3197	0.93	0.7246	0.1012	0.4259	1.7368	PREDICTED: probable bifunctional methylthioribulose-1-phosphate dehydratase/enolase-phosphatase E1 [Jatropha curcas]
Unigene00104[illegible]	0	0	0	1.0017	1.5967	0	0	1.8567	0	Cysteine synthase [Ectocarpus siliculosus]

Unigene00105[illegible]	0.177	1.7345	0.0615	0.3216	0.1743	1.1604	0.698	0.4007	0.0319	PREDICTED: zinc finger CCCH domain-containing protein 16-like isoform X1 [Citrus sinensis]
Unigene00109[illegible]	0.4737	0	0.1975	1.2785	0	0.1147	0	1.2867	0	2-oxoglutarate dehydrogenase, E1 component [Chondrus crispus]
Unigene00109[illegible]	0	0.4846	0.2629	0.1745	0.1987	0.5087	0	0.1427	0	PREDICTED: transcription factor bHLH106-like [Fragaria vesca subsp. vesca]
Unigene00109[illegible]	23.693	0.834	0.0628	1.0013	0.8194	2.2984	0.1783	0.5117	0.7814	Duplicated homeodomain-like superfamily protein [Theobroma cacao]
Unigene00112[illegible]	0.1487	0.1714	0.8264	1.2346	0.8199	0.8996	0.0489	0.2243	1.8732	leafy cotyledon 1-like protein [Litchi chinensis]
Unigene00114[illegible]	6.0606	9.1256	3.4809	8.8593	1.9003	11.3766	5.2432	4.6195	0.9351	PREDICTED: zeatin O-glucosyltransferase-like [Nicotiana tomentosiformis]
Unigene00115[illegible]	0.243	0	0	0.1261	2.0824	0.0735	0	0.2063	1.2468	indoleamine 2,3-dioxygenase 2 [Monoraphidium neglectum]
Unigene00117[illegible]	2.2319	2.7084	0.2449	2.5608	5.0443	3.886	2.0073	1.3295	6.2592	terminal flower 1 protein [Dimocarpus longan]
Unigene00119[illegible]	0	0	2.8512	0	0	0	1.2064	0	0	flowering locus T [Litchi chinensis]
Unigene00119[illegible]	1.6568	1.4027	0.5397	0.4837	2.8148	1.5039	0.638	0.7618	4.0543	SCL domain class transcription factor [Theobroma cacao]
Unigene00121[illegible]	0.7123	1.4565	0.1261	1.8557	0.0953	2.9768	0.636	6.2673	6.2712	GH3 [Dimocarpus longan]
Unigene00121[illegible]	12.8154	68.3271	14.5476	104.719	37.8321	79.2938	10.4576	181.1885	5.0921	PREDICTED: cyclic dof factor 3-like [Citrus sinensis]
Unigene00121[illegible]	32.0163	42.5056	18.8545	47.9936	52.5008	42.1753	14.3382	87.079	45.94	PREDICTED: transcription factor MYB1R1-like [Citrus sinensis]
Unigene00121[illegible]	0.3912	0.7141	0.4418	2.0138	1.156	0.947	0.1928	2.5092	1.2501	PREDICTED: transcription factor MYB1R1-like [Citrus sinensis]
Unigene00122[illegible]	3.5344	5.908	0.1612	5.2741	1.3576	2.513	0	16.5256	0.1432	PREDICTED: transcription factor MYB44-like [Cucumis sativus]
Unigene00122[illegible]	9.2411	23.8681	2.6179	11.632	14.2706	8.4953	2.3807	11.8803	15.302	WRKY transcription factor 16 [Dimocarpus longan]
Unigene00123[illegible]	0.3041	0.4207	7.3535	0.1263	0.3594	0.6624	2.5781	1.239	5.5505	PREDICTED: auxin-responsive protein IAA14 [Prunus mume]
Unigene00132[illegible]	2.1815	1.7914	0	1.458	1.0596	1.1212	0	1.4205	1.7752	TFL1-1 [Litchi chinensis]

Unigene00134[illegible]	0.1213	0	0	0.8813	0	0	0	1.7844	0	PREDICTED: aldehyde dehydrogenase family 3 member H1-like isoform X2 [Fragaria vesca subsp. vesca]
Unigene00135[illegible]	1.6432	1.6237	3.7246	1.1643	1.2947	0.8839	4.0113	1.1513	0.169	NAC domain protein, IPR003441 [Theobroma cacao]
Unigene00137[illegible]	277.0283	612.7368	38.8928	662.0995	31.8315	424.0057	77.1567	1802.3363	799.7306	PREDICTED: zinc finger protein ZAT10-like [Citrus sinensis]
Unigene00138[illegible]	13.696	7.129	0.9517	7.1519	18.394	8.4888	2.7822	9.5824	10.6238	PREDICTED: ethylene-responsive transcription factor ERF023-like [Cicer arietinum]
Unigene00140[illegible]	0.3607	0.9565	0.9025	1.0111	0.0853	1.0478	1.4581	0.1633	4.4808	PREDICTED: transcription factor BEE 3-like [Citrus sinensis]
Unigene00140[illegible]	1.6111	0.5715	0.2584	0.5146	2.3435	0.8999	0.1222	1.6832	1.6732	Nuclear transcription factor Y subunit B-5 [Theobroma cacao]
Unigene00141[illegible]	0.5068	0	0	1.4468	0	0	0	2.2944	0	3-ketoacyl-CoA thiolase 2, peroxisomal -like protein [Gossypium arboreum]
Unigene00143[illegible]	0.8614	0	3.3468	0.1626	0	0	0.6948	0	1.1842	PREDICTED: DELLA protein GAI-like [Citrus sinensis]
Unigene00143[illegible]	0	1.1325	0.2793	0.7417	0	0	0.8804	0	0.5788	PREDICTED: agamous-like MADS-box protein AGL11-like isoform X1 [Citrus sinensis]
Unigene00144[illegible]	0.5241	1.4905	2.1125	1.3058	0.3717	0.5075	1.6879	0.791	0.7548	PREDICTED: NAC domain-containing protein 86 isoform X1 [Populus euphratica]
Unigene00145[illegible]	0.0898	0	0.1248	0.1864	0.0707	0	0.0295	0.2371	0	PREDICTED: two-component response regulator ARR11-like isoform X1 [Citrus sinensis]
Unigene00145[illegible]	0.6525	0.6997	0.7276	0.5041	0	0.2755	0.2693	0.5496	0.0328	PREDICTED: two-component response regulator ARR11-like isoform X1 [Citrus sinensis]
Unigene00146[illegible]	0.4772	0.5502	0	0.0619	0.1763	0.5415	0	0	0	Duplicated homeodomain-like superfamily protein [Theobroma cacao]
Unigene00146[illegible]	1.1431	1.3178	0.7692	1.2768	0.1368	1.0508	3.9943	0.0164	0.1875	PREDICTED: protein FEZ-like [Citrus sinensis]

Unigene00148	0.3694	0.131	0	0.2949	2.8207	0.8253	0	2.1224	0.3069	PREDICTED: probable indole-3-pyruvate monooxygenase YUCCA3 [Musa acuminata subsp. malaccensis]
Unigene00149	0	0.7613	0.8719	0.457	0.1041	0.1598	3.5588	0.0997	0	PREDICTED: acyl-coenzyme A oxidase 4, peroxisomal isoform X2 [Vitis vinifera]
Unigene00150	1.6725	0	0.5811	0	0	0	1.399	0.3442	4.1602	PREDICTED: LOW QUALITY PROTEIN: transcription factor MYB113-like [Citrus sinensis]
Unigene00156	9.375	24.5162	7.1068	54.9513	7.3386	42.9287	3.5609	118.9506	20.8503	PREDICTED: transcription factor DIVARICATA-like [Citrus sinensis]
Unigene00159	256.376	197.9052	25.8025	231.0403	6.6665	162.2409	42.3574	876.5835	503.7474	PREDICTED: ethylene-responsive transcription factor ERF017-like [Citrus sinensis]
Unigene00159	0.8058	5.0189	1.6915	6.9242	0.3439	9.4001	4.0381	14.5539	10.2262	Zinc finger family protein [Theobroma cacao]
Unigene00160	1.0993	0	0.7414	0.8727	0.7643	2.4261	0.0637	0.5855	0.8381	PREDICTED: auxin-responsive protein IAA1-like [Citrus sinensis]
Unigene00167	5.4479	18.9176	2.4091	10.8995	20.7989	9.9501	1.8879	33.1837	8.5231	Ethylene-responsive transcription factor RAP2-3-like protein [Gossypium arboreum]
Unigene00172	0.7186	1.2426	0.1873	2.3311	0.8493	0.2174	0	0	0.5821	PREDICTED: salicylate carboxymethyltransferase isoform X1 [Vitis vinifera]
Unigene00172	0	0	0	1.7748	0	0.1592	0	1.3396	0.8523	1-aminocyclopropane-carboxylate synthase [Dimocarpus longan]
Unigene00173	0.113	0.152	0.432	0.2738	0.1336	0.4446	0.3157	0.064	0.1628	PREDICTED: protein FAR-RED ELONGATED HYPOCOTYL 3-like isoform X1 [Citrus sinensis]
Unigene00174	0.0694	1.2404	0	0.5765	0	2.3104	0	0.3928	0	PREDICTED: 4-coumarate--CoA ligase 2-like [Elaeis guineensis]
Unigene00175	0.6445	0.878	0.2443	0.3649	0	0.0355	1.0685	0.0663	0.5695	Squamosa promoter binding protein-like 4 [Theobroma cacao]

Unigene001840	0.5062	0.667	0	0.1501	1.8803	0.0875	0	0.7366	1.0154	PREDICTED: bifunctional aspartokinase/homoserine dehydrogenase, chloroplastic-like [Nicotiana tomentosiformis]
Unigene001884	0	0.2034	0	0.3663	0.278	1.7082	0	2.1299	0	PREDICTED: similar to Zinc finger protein 271 (Zinc finger protein 7) (HZF7) (Zinc finger protein ZNFphex133) (Epstein-Barr virus-induced zinc finger protein) (ZNF-EB) (CT-ZFP48) (Zinc finger protein [Bathycoccus prasinos]
Unigene001912	1.9853	1.1443	1.1381	3.9157	6.0417	3.0034	0.1957	8.5382	3.9668	C2H2-type zinc finger protein 1 [Populus trichocarpa]
Unigene001933	2.8864	0.8781	0.7104	1.5189	1.6819	0.7763	0.7904	5.0362	4.1999	PREDICTED: dehydration-responsive element-binding protein 2D-like [Citrus sinensis]
Unigene001962	1.1459	0.7926	2.0703	0.1586	0.7223	1.4793	1.1297	2.248	0.9901	amidase [Cucumis melo subsp. melo]
Unigene001980	0.9287	0	2.6015	0	0	0	1.7166	0.0657	0	PREDICTED: 9-cis-epoxycarotenoid dioxygenase NCED6, chloroplastic-like [Citrus sinensis]
Unigene00201[illegible]	0	0.4074	0	0.0367	0	0.2566	0.1394	0.08	0	PREDICTED: zinc finger CCCH domain-containing protein ZFN-like [Prunus mume]
Unigene002010	1.0308	3.1688	0.5116	2.1401	1.3923	0.7129	0.1936	1.1111	5.0364	PREDICTED: zinc finger protein 2-like [Populus euphratica]
Unigene002029	4.5433	7.4615	1.8255	3.9786	2.3098	5.345	9.2082	1.896	3.7283	PREDICTED: myb family transcription factor APL isoform X2 [Vitis vinifera]
Unigene002029	34.388	4.6391	8.9991	8.0509	16.9485	6.8626	2.4524	10.9307	20.3488	PREDICTED: LOB domain-containing protein 33 [Jatropha curcas]
Unigene002045	3.9462	3.2642	1.6035	2.5922	0.8959	3.13	2.6156	2.0439	0.7224	WRKY protein [Salvia miltiorrhiza]
Unigene002048	0.2317	0	0	1.4434	0	0.1402	0	1.3114	0.2503	1,2-dihydroxy-3-keto-5-methylthiopentene dioxygenase [Medicago truncatula]

Unigene002128	5.5992	1.6496	7.0035	0.5812	2.2058	1.6565	5.9492	1.1266	3.1581	PREDICTED: flowering-promoting factor 1-like [Malus domestica]
Unigene002130	0.1283	0	0	0.9321	0	0	0	1.0162	0.1385	enoyl-CoA hydratase [Galdieria sulphuraria]
Unigene002142	0.3476	1.2022	0	0.1203	0	1.4023	0	0	0.1251	PREDICTED: 4-coumarate--CoA ligase-like 7 [Cucumis sativus]
Unigene002155	1.2461	1.5961	1.4612	1.024	1.3499	2.2202	0.6142	3.8587	0.7103	BZIP transcription factor family protein isoform 1 [Theobroma cacao]
Unigene002187	0	3.0069	0	3.3845	0.1541	0.7103	1.0285	0.0738	0	PREDICTED: auxin-induced protein X10A-like isoform X1 [Citrus sinensis]
Unigene002237	4.4827	4.4642	5.5281	2.0755	1.6416	4.5338	5.4359	1.0957	3.9552	Homeobox protein 33 isoform 1 [Theobroma cacao]
Unigene002240	0.7712	1.3336	0	0.934	0	0.1556	0	3.7095	0.2776	PREDICTED: 2-oxoglutarate dehydrogenase, mitochondrial [Tarenaya hassleriana]
Unigene002275	3.497	1.3146	9.9842	2.6832	12.4887	1.4721	1.7987	8.604	4.8033	PREDICTED: ocs element-binding factor 1-like [Citrus sinensis]
Unigene002277	4.7717	6.8558	9.0899	5.2218	2.6392	4.6371	9.246	2.2081	3.7574	PREDICTED: zinc finger protein WIP2 [Vitis vinifera]
Unigene002298	2.4029	0.3957	0.0716	0.2138	1.5822	1.2463	0.203	1.8648	1.2976	PREDICTED: cytokinin dehydrogenase 3-like [Citrus sinensis]
Unigene002312	2.6905	0.8589	0.1294	0.2578	0	1.1522	1.8363	0.2811	0.5365	PREDICTED: B3 domain-containing transcription factor VRN1-like isoform X2 [Citrus sinensis]
Unigene002333	0	0	0	0.6407	0	0	0	0.3992	0	LFY protein [Litchi chinensis]
Unigene002337	0.4286	0.6353	1.6276	1.2713	1.0856	0.9264	0.2717	0.6931	0.0992	PREDICTED: transcription factor HBP-1b(c1)-like isoform X2 [Citrus sinensis]
Unigene002370	13.6187	10.116	7.5367	11.769	14.9339	8.1137	7.4396	15.1355	18.5002	LOB domain-containing protein 31 [Theobroma cacao]
Unigene002373	6.2445	17.7635	0.1127	12.5719	4.2173	13.2175	0.3731	2.2946	0.4672	PREDICTED: agamous-like MADS-box protein AGL19-like isoform X5 [Citrus sinensis]

Unigene00239[illegible]	3.5826	2.6433	0.6722	1.4876	0	3.2953	0.7064	0.6488	0 PREDICTED: cystathionine gamma-synthase 1, chloroplastic [Jatropha curcas]
Unigene00240[illegible]	0	0.4991	1.2634	0	0	0.4191	0.6828	0.196	0.0935 PREDICTED: malate dehydrogenase, cytoplasmic-like isoform X1 [Citrus sinensis]
Unigene00242	0.1925	1.3041	0	0.3748	0.2276	0.9613	0.9491	0.4086	0.026 PREDICTED: homeobox protein ATH1-like [Citrus sinensis]
Unigene00242[illegible]	0.3388	0.5208	0	0.3517	1.001	1.6401	0	0.7669	0.4879 acetyl- acetyltransferase [Nannochloropsis gaditana]
Unigene00243[illegible]	0	1.0889	0	0.8579	0	0	0	2.9397	0.1275 4-hydroxyphenylpyruvate dioxygenase [Galdieria sulphuraria]
Unigene00243[illegible]	0	0.6304	0	0.3784	1.1848	0	0	1.4441	0.689 --
Unigene00245	0	0.1341	0	1.5695	0	0	0.1147	1.9744	0 PREDICTED: LOW QUALITY PROTEIN: catalase-B-like, partial [Elaeis guineensis]
Unigene00245[illegible]	1.0148	0.8913	0.5037	1.2039	0.6853	1.1697	0.1906	0.9298	1.618 probable bifunctional methylthioribulose-1-phosphate dehydratase/enolase-phosphatase E1 [Zea mays]
Unigene00248[illegible]	0	0.7223	0	0.0813	0	0.6635	0	0.0886	0 PREDICTED: auxin-responsive protein IAA2-like [Populus euphratica]
Unigene00248[illegible]	1.1738	3.7748	1.1591	2.4692	0.2921	2.6918	1.5836	1.0838	0.0667 PREDICTED: auxin-responsive protein IAA26-like isoform X1 [Citrus sinensis]
Unigene00249	0.3578	2.0627	0.9325	0.8173	0.8036	0.6929	2.1873	0.2025	0.0773 AGAMOUS-like protein [Mangifera indica]
Unigene00250[illegible]	2.811	2.3828	15.4686	0.8582	1.5145	2.5515	9.9844	1.1696	7.5008 bZIP family protein [Populus trichocarpa]
Unigene00253[illegible]	1.8245	1.0955	2.4564	0.947	0.3594	0.46	1.4989	3.1405	0.1642 PREDICTED: transcription factor MYB39 [Vitis vinifera]
Unigene00255[illegible]	2.0235	3.913	0.3062	6.3356	1.8901	3.634	0.1287	2.401	4.5473 PREDICTED: nuclear transcription factor Y subunit B-9-like [Citrus sinensis]
Unigene00256[illegible]	12.1913	6.8331	11.9177	3.2163	3.7016	3.5868	11.9025	1.3341	3.7102 PREDICTED: aspartate aminotransferase, mitochondrial-like [Citrus sinensis]

Unigene00257	0.1574	0.5898	0	0	1.0698	3.2864	0	0	1.9978	cytochrome P450 family 806A-CYP806A1 [Chondrus crispus]
Unigene002587	9.9846	14.224	3.2647	6.5815	1.7499	7.9488	16.4117	1.5468	1.6197	PREDICTED: zinc finger protein CONSTANS-LIKE 13-like [Citrus sinensis]
Unigene002588	0	0	5.0169	0	0	0	1.336	0.1058	0	PREDICTED: abscisic acid 8'-hydroxylase 4-like [Populus euphratica]
Unigene002589	0.0609	0.7376	1.0163	1.3284	0.072	1.2906	1.2315	0.8276	0.1974	PREDICTED: zinc finger protein CONSTANS-LIKE 1-like isoform X3 [Citrus sinensis]
Unigene002615	3.0975	3.4134	2.5521	4.4095	6.8775	5.6783	2.0433	8.4292	5.3504	PREDICTED: homeobox-leucine zipper protein ANTHOCYANINLESS 2-like isoform X1 [Citrus sinensis]
Unigene00262	0	0	0	2.6193	0	0	0	1.3439	0	PREDICTED: nuclear transcription factor Y subunit A-3-like [Elaeis guineensis]
Unigene00263	1.3295	1.2488	0.7185	0.9201	0.5237	0.7151	1.6504	0	0	PREDICTED: transcription factor WER-like isoform X1 [Citrus sinensis]
Unigene00263	4.8301	3.1364	5.8565	1.6782	3.0057	3.3881	4.8977	1.1379	6.0685	WOX1b [Populus tomentosa]
Unigene00264	3.2581	9.1409	3.6966	8.71	8.3488	5.8276	2.473	4.1118	1.7751	PREDICTED: ethylene-responsive transcription factor ERF113 [Nelumbo nucifera]
Unigene002644	3.4314	0.5651	2.6399	0.5089	0	2.6697	0	6.2416	4.8533	PREDICTED: ethylene-responsive transcription factor 1B [Gossypium raimondii]
Unigene00264	88.8821	83.0957	41.017	113.6992	59.1912	70.6775	14.3695	439.7918	103.2128	PREDICTED: NAC domain-containing protein 2-like [Citrus sinensis]
Unigene00265	0.0353	0.4074	0	0.7337	0	0.4277	0.5923	0.16	0.458	PREDICTED: WUSCHEL-related homeobox 8 [Vitis vinifera]
Unigene002655	60.4086	96.5418	20.7737	87.0316	70.1002	72.7357	21.1735	111.4346	96.0297	PREDICTED: auxin-responsive protein IAA16-like [Populus euphratica]
Unigene00266	1.5517	5.6776	0.0703	4.7273	1.9535	9.7163	0	0.2291	0.0729	3-hydroxyacyl-coenzyme A dehydrogenase [Phaeodactylum tricornutum CCAP 1055/1]

Unigene00267	79.825	48.1846	36.6132	89.8682	62.2488	74.806	20.584	162.924	74.6763	DNA binding with one finger 5 family protein [Populus trichocarpa]
Unigene00268	0	0	2.4495	0	0	0	4.1458	0	0	APETALA 1-2 [Dimocarpus longan]
Unigene00268	0.225	0.7135	176.5162	0.3504	0.4655	0.2724	296.7635	0.191	0.3038	APETALA 1-1 [Dimocarpus longan]
Unigene00269	0.396	1.6955	0	0.4698	0.6017	1.5062	0	0.3201	0.1222	4-coumarate--CoA ligase-like protein 5 [Aegilops tauschii]
Unigene00270	4.0154	4.1767	2.9721	3.2043	1.0048	3.3401	2.3339	1.8208	1.003	ethylene receptor [Dimocarpus longan]
Unigene00270	10.8591	8.4924	2.9062	5.1184	1.5609	7.175	11.747	0.7308	1.2046	Far-red impaired responsive family protein isoform 1 [Theobroma cacao]
Unigene00270	117.0744	73.9588	12.3232	68.6164	65.5083	47.2612	25.4043	70.35	29.408	PREDICTED: two-component response regulator ARR6-like isoform X1 [Nicotiana sylvestris]
Unigene00271	14.3037	31.6032	2.5113	15.9075	2.1805	22.7227	5.4993	7.0258	8.0644	PREDICTED: myb-related protein 330-like [Prunus mume]
Unigene00274	309.2124	305.8698	93.8082	433.409	183.6834	335.2322	56.4671	926.4648	293.0876	RecName: Full=S-adenosylmethionine synthase; Short=AdoMet synthase; AltName: Full=Methionine adenosyltransferase; Short=MAT [Litchi chinensis]
Unigene00274	0.3938	1.3945	8.8551	3.037	4.2223	4.5961	2.0799	6.0492	1.4584	gibberellin 3 beta-hydroxylase family protein [Populus trichocarpa]
Unigene00277	7.9338	23.7464	0.3082	37.8805	6.8724	24.184	1.1659	29.6433	4.8434	PREDICTED: myb-related protein 3R-1-like [Citrus sinensis]
Unigene00277	2.0488	6.8947	1.2876	6.5681	13.104	3.7923	0.6832	5.3025	8.0215	WRKY transcription factor 47-2 [Dimocarpus longan]
Unigene00278	113.0085	112.5963	27.8618	265.5123	89.0635	147.704	38.569	447.59	319.0586	PREDICTED: transcription factor MYB44-like [Populus euphratica]
Unigene00278	0.7377	0	0	2.6165	0	0.8928	0	1.1133	0	Cysteine synthase [Ectocarpus siliculosus]
Unigene00278	0.1115	2.3783	0	1.0997	0.659	3.779	0.1099	0.2524	0.3613	cytochrome P450 78A3p family protein [Populus trichocarpa]

Unigene00279	59.5896	68.9045	25.5018	86.9397	72.0176	67.5172	26.0896	97.0336	67.9883	PREDICTED: squamosa promoter-binding-like protein 7 [Populus euphratica]
Unigene00279	1.4994	0	0	3.9741	0	0.3475	0	0.5777	0	--
Unigene00279	1.8416	0.9614	4.2738	0.9378	0.4928	2.9858	0.3768	0.3539	3.3026	ethylene response factor 10 [Actinidia deliciosa]
Unigene00279	0.1925	0.3328	0.3762	0.7118	1.4502	0.1747	0.9725	0.1089	0.3378	PREDICTED: transcription factor TCP4-like isoform X1 [Citrus sinensis]
Unigene00279	1.6048	3.0423	1.6356	0.8144	1.2644	1.6833	1.4414	1.1705	2.3881	PREDICTED: indole-3-pyruvate monooxygenase YUCCA2-like [Citrus sinensis]
Unigene00281	8.2649	7.2672	1.4602	12.0698	0.4967	7.0358	11.7386	3.4087	6.8842	PREDICTED: auxin-induced protein 10A5 [Fragaria vesca subsp. vesca]
Unigene00282	0.9967	0.2089	0.2833	0.5643	4.7117	0.2193	1.1612	0.8717	1.8594	PREDICTED: cytokinin dehydrogenase 3-like [Citrus sinensis]
Unigene00285	6.4951	5.3875	2.1879	8.1814	3.4637	7.3815	1.3275	17.9295	6.7585	PREDICTED: transcription factor DIVARICATA-like [Jatropha curcas]
Unigene00287	0.5934	0.3909	0	0.9974	1.9704	1.1628	0	2.0469	0.7325	PREDICTED: aldehyde dehydrogenase family 3 member H1 [Phoenix dactylifera]
Unigene00288	0.8621	0.2795	0	5.9845	0	1.3041	0	1.3415	0	S-Adenosylmethionine decarboxylase [Ectocarpus siliculosus]
Unigene00289	3.6204	4.7494	2.1688	3.1967	5.0165	2.77	0.6154	4.0504	5.5733	PREDICTED: LOB domain-containing protein 4-like [Gossypium raimondii]
Unigene00289	1.1345	0.2474	0.1278	2.069	0	0.7422	0	4.7544	0.5961	--
Unigene00291	0.1015	1.0534	0.0529	4.5846	0.48	1.5974	0.1001	2.9876	0	gibberellin 20-oxidase-like protein2 [Ipomoea nil]
Unigene00294	41.538	47.9095	6.8762	33.9264	17.0953	35.9643	6.2051	162.3627	96.3738	PREDICTED: serine acetyltransferase 1, chloroplastic-like [Cicer arietinum]
Unigene00294	0.2162	0.1662	5.0329	0.1496	0	0	14.2803	0	0	MADS domain transcription factor [Camellia japonica]
Unigene00295	21.1502	13.7273	13.8884	10.225	1.9535	15.2277	30.6276	3.2833	6.0115	Phospholipase A21 [Theobroma cacao]

Unigene00296	15.7392	17.4303	9.3654	17.3679	22.1169	20.7735	5.4369	23.4942	41.0264	PREDICTED: G-box-binding factor 4-like [Citrus sinensis]
Unigene00296	0.9201	2.4529	0	1.8506	1.0195	3.7583	0	3.5797	0.8074	BnaC02g30540D [Brassica napus]
Unigene00299	0.409	0.1347	52.1884	0.1819	0.3452	0.3535	26.2925	0.9257	0.5048	MADS-13 [Gossypium hirsutum]
Unigene00300	64.5458	47.4611	6.2544	31.7068	4.6785	30.7124	10.7217	80.3151	62.2881	Cys2/His2-type zinc finger protein [Citrus trifoliata]
Unigene00301	0.1523	1.2289	0	0.7378	0.36	2.5189	0	0	0	malate dehydrogenase [Selaginella moellendorffii]
Unigene00301	49.4076	64.1953	52.8343	77.1609	108.7764	71.1356	46.5837	93.5284	112.7318	aux/IAA protein [Populus tremula x Populus tremuloides]
Unigene00301	195.9301	93.3496	12.0417	82.4691	5.9747	80.0764	33.5001	193.2042	106.6456	WRKY transcription factor 2-7 [Dimocarpus longan]
Unigene00302	3.4809	5.6099	10.163	5.4203	1.3853	4.6427	18.2614	1.1658	2.5896	PREDICTED: auxin transporter-like protein 3 [Gossypium raimondii]
Unigene00303	3.8207	4.4278	1.3415	2.0457	0.6417	3.8452	10.4671	0.6828	0.5213	Sequence-specific DNA binding transcription factors [Theobroma cacao]
Unigene00304	11.0382	0.4486	0.1844	0.5142	0.1673	1.3702	0.7673	2.1222	1.5285	PREDICTED: transcription factor bHLH92-like [Citrus sinensis]
Unigene00306	5.0855	3.7794	6.651	1.7597	3.6478	2.4774	11.9031	0.3801	1.4682	PREDICTED: cytochrome P450 724B1-like [Pyrus x bretschneideri]
Unigene00306	4.0753	16.5242	58.649	12.5163	5.6468	10.2384	90.6583	3.1491	5.6765	APETALA1 [Litchi chinensis]
Unigene00306	0	0.4178	0	0.1254	0.1904	0.5361	0.3176	0.1367	0	bZIP transcription factor family protein 2 [Camellia sinensis]
Unigene00306	0.8555	1.726	0.0811	0.2422	0	1.2237	0.7092	0.2641	0.042	bZIP transcription factor family protein 2 [Camellia sinensis]
Unigene00307	3.5854	5.0258	7.1772	5.2234	2.8651	5.572	6.1053	6.0869	2.0683	Dof-type zinc finger DNA-binding family protein [Theobroma cacao]
Unigene00313	0.4997	0.288	0.5209	1.5562	0.1476	0.1512	0.7389	1.1311	1.3493	transport inhibitor response 1 protein [Medicago truncatula]
Unigene00315	6.2397	2.6033	5.0173	3.0227	7.3393	2.8768	2.109	5.2124	10.5903	Trihelix transcription factor GT-4 -like protein [Gossypium arboreum]

Unigene00316	18.2155	23.1867	4.4039	15.8364	2.5116	18.31	12.271	7.2156	12.3509	PREDICTED: transcription factor bHLH113 isoform X1 [Prunus mume]
Unigene00317	0	0.2604	0	0.7034	2.0821	1.9681	0.3563	3.0675	1.2685	PREDICTED: cystathionine beta-lyase, chloroplastic isoform X3 [Nicotiana tomentosiformis]
Unigene00317	2.3116	1.0101	2.4677	0.3096	0.4406	0.4061	6.4689	0.2954	1.1879	PREDICTED: zinc finger protein WIP2 [Jatropha curcas]
Unigene00317	0.355	0.1637	0.7401	0	0.0839	0.3437	0.49	0	0.2684	PREDICTED: zinc finger protein WIP2-like [Sesamum indicum]
Unigene00317	0	0.2852	1.9338	1.1556	1.3156	0.5988	0	0	0	PREDICTED: adenylate isopentenyltransferase 5, chloroplastic [Cicer arietinum]
Unigene00318	2.8727	2.1526	1.0854	3.0194	5.9839	3.5636	1.7346	5.4053	5.1583	PREDICTED: LOB domain-containing protein 11-like [Citrus sinensis]
Unigene00318	3.8386	1.6926	6.8406	2.0383	1.9025	1.7769	3.9935	3.7238	2.9996	PREDICTED: ent-kaurene oxidase, chloroplastic-like [Citrus sinensis]
Unigene00319	1.1037	0.3563	60.6281	0.3208	0.3652	0	40.4331	0.2498	0.5484	PREDICTED: probable transcription factor KAN4 [Fragaria vesca subsp. vesca]
Unigene00320	1.2229	4.9154	2.7906	19.1801	2.9298	10.6006	0.0652	48.4816	0.9282	PREDICTED: homeobox-leucine zipper protein ATHB-12-like isoform X1 [Citrus sinensis]
Unigene00320	1.8083	38.291	11.6065	48.7558	174.962	16.8167	14.5427	15.5647	89.1721	pathogenesis-related gene 1-like protein PR1-1 [Dimocarpus longan]
Unigene00322	3.5131	3.647	1.319	3.1457	0.8068	4.9772	2.0025	1.1495	0.5755	PREDICTED: B3 domain-containing transcription factor VRN1-like [Citrus sinensis]
Unigene00323	0	0.471	0	0.6665	0	0	0	4.6242	3.9716	--
Unigene00326	41.1055	48.1443	44.4739	47.0884	22.4714	44.4339	48.1329	37.8382	21.9051	terminal flower 2-1 [Dimocarpus longan]
Unigene00328	0.9794	1.594	0.9608	1.0167	0.8171	0.0697	0.3976	0.3912	0.56	IAA30 - auxin-responsive Aux/IAA family member [Zea mays]

Unigene003283	1.3207	1.8271	2.2026	0.6855	0.6244	1.5984	3.7758	1.0463	0.5706	PREDICTED: tRNA dimethylallyltransferase 9-like [Citrus sinensis]
Unigene003290	63.6373	24.919	1.1659	29.6254	0.2858	48.0401	8.2854	35.5159	86.8221	CBF/DREB-like transcription factor 1 [Citrus trifoliata]
Unigene003300	3.7942	4.121	3.0723	7.4542	3.3353	7.0587	1.422	13.273	6.401	homeobox-leucine zipper family protein [Populus trichocarpa]
Unigene003310	1.0936	0.4034	17.9655	0.3633	2.068	0.4765	8.4312	0.4704	7.0636	Squamosa promoter binding protein-like 3 isoform 2, partial [Theobroma cacao]
Unigene00333	34.2956	39.7695	12.1262	31.6227	4.8083	31.537	26.12	10.8074	5.2294	PREDICTED: protein FAR1-RELATED SEQUENCE 2 [Populus euphratica]
Unigene003332	6.4381	4.8302	3.3021	4.7738	3.5025	2.2262	7.2033	3.3541	18.4878	Auxin-induced protein 10A5 [Glycine soja]
Unigene003332	0.7901	0.4315	0.1084	3.3025	0	0.6543	0	2.9652	0.2695	PREDICTED: LOW QUALITY PROTEIN: catalase-B-like, partial [Elaeis guineensis]
Unigene003338	2.058	6.686	0	4.195	6.368	3.3964	2.287	0.847	7.1532	PREDICTED: homeobox-leucine zipper protein HAT22-like [Pyrus x bretschneideri]
Unigene003338	4.7767	1.5946	9.1698	3.0633	2.8336	4.4419	0.7636	3.5068	0.996	PREDICTED: transcription factor MYC2-like [Citrus sinensis]
Unigene003344	11.9454	9.0894	0.5222	8.9512	13.588	13.5315	2.6516	3.9992	12.7309	Homeobox-leucine zipper protein ATHB-51 [Theobroma cacao]
Unigene003370	91.9702	256.7227	46.7726	355.7695	179.0658	244.0153	44.9944	567.2968	425.9406	PREDICTED: zinc finger CCCH domain-containing protein 20 [Jatropha curcas]
Unigene003384	57.5698	58.5906	45.6775	64.1355	92.137	57.8989	30.5353	125.9192	133.184	PREDICTED: DELLA protein GAIP-B-like [Citrus sinensis]
Unigene003384	32.979	79.6676	50.0452	99.3548	184.7435	62.4894	20.9505	157.942	252.4829	CRY [Pisum sativum]
Unigene003385	25.2186	1.5952	0.2163	1.7237	1.4718	2.8051	0.0682	13.0378	20.4751	PREDICTED: ethylene-responsive transcription factor 2-like [Prunus mume]
Unigene003380	5.2799	3.2725	2.1896	0.884	1.2078	2.2675	26.6416	0.514	0.3986	PREDICTED: transcription repressor MYB5-like [Jatropha curcas]
Unigene003392	0.7027	0.4704	0.1418	3.8827	0	1.5912	0	1.2828	0	PREDICTED: 3-ketoacyl-CoA thiolase 2, peroxisomal-like isoform X1 [Glycine max]

Unigene003395	9.2916	5.1574	10.1206	2.6793	0.9296	1.7255	7.9969	2.0031	4.5134	Homeobox-leucine zipper ATHB-20 -like protein [Gossypium arboreum]
Unigene003400	4.6904	4.5526	1.1671	2.6387	0.6438	3.8272	0.1193	6.6614	3.726	PREDICTED: scarecrow-like protein 30 [Gossypium raimondii]
Unigene003407	1.632	1.5125	3.3356	2.2589	1.0212	4.144	3.8488	1.3038	0.553	Basic helix-loop-helix DNA-binding superfamily protein [Theobroma cacao]
Unigene003417	1.313	7.1404	0	5.2445	2.3276	5.3889	0.1126	0.7753	0.8632	flowering locus C [Dimocarpus longan]
Unigene003417	28.7558	31.5501	0.8958	50.9868	52.8922	39.7224	0.6517	35.3886	41.4468	flowering locus C [Dimocarpus longan]
Unigene003424	1.951	1.8652	0.5952	0.8068	4.5929	1.8237	1.5794	0.2872	1.0451	PREDICTED: indole-3-pyruvate monooxygenase YUCCA6-like [Gossypium raimondii]
Unigene003452	9.5713	63.8316	4.523	150.5556	21.8326	73.9741	7.6757	150.2824	21.8269	PREDICTED: heat stress transcription factor C-1 [Populus euphratica]
Unigene003450	0.8275	1.0902	0	0.4909	1.8162	0.9538	0	1.4271	1.5747	PREDICTED: 12-oxophytodienoate reductase 2-like [Prunus mume]
Unigene003460	9.9824	15.5625	3.5123	5.3999	2.5139	12.1578	35.1704	3.4085	3.1162	PREDICTED: B3 domain-containing protein Os01g0723500-like [Prunus mume]
Unigene003465	1.0426	4.5231	0.7436	5.5825	5.545	4.6821	1.0008	4.658	2.6374	PREDICTED: nuclear transcription factor Y subunit B-3 [Vitis vinifera]
Unigene003468	21.9876	23.5567	14.3302	23.4603	40.3427	21.6533	7.8443	40.3387	32.9525	SAUR family protein [Theobroma cacao]
Unigene003469	5.4272	4.5621	9.3104	1.252	1.6926	1.8245	13.636	0.0853	2.361	PREDICTED: auxin-induced protein 15A [Vitis vinifera]
Unigene003474	8.9604	15.4808	1.2051	5.426	0.8683	11.3109	12.9416	2.2763	1.4309	PREDICTED: heat stress transcription factor A-8-like [Citrus sinensis]
Unigene003474	7.8189	7.8409	0.6699	5.337	1.2026	10.7593	5.5436	1.6365	2.8921	PREDICTED: heat stress transcription factor A-8-like, partial [Gossypium raimondii]
Unigene003492	0.1022	0.0589	101.6193	0.1856	0	0.0618	55.8634	0	0	PREDICTED: developmental protein SEPALLATA 1-like [Citrus sinensis]

Unigene00349	1.1818	1.2602	5.9742	1.8708	2.8632	1.7163	10.2231	0.2341	1.3402	PREDICTED: NAC domain-containing protein 19-like [Prunus mume]
Unigene00350	6.0942	2.9332	3.3151	2.9821	5.893	2.7317	1.9824	3.6229	6.8038	PREDICTED: capsanthin/capsorubin synthase, chromoplast-like [Prunus mume]
Unigene00351	0.6143	1.1182	1.3481	0.6377	1.2228	1.3305	2.0081	0.2928	0.4191	PREDICTED: serine/threonine-protein kinase SAPK2 [Eucalyptus grandis]
Unigene00352	0.9943	0.0498	0	1.077	0	0.3662	0	2.8378	1.2607	aspartate aminotransferase [Petalonia fascia]
Unigene00353	27.0831	29.741	41.6751	32.3824	75.5266	28.144	21.2027	53.947	92.4175	MYB1 [Gossypium hirsutum]
Unigene00354	12.3924	3.4092	20.8761	2.6476	0.9616	3.4086	23.8322	3.7897	3.1772	Aldehyde dehydrogenase family 2 member B7, mitochondrial [Gossypium arboreum]
Unigene00354	0.8475	0.2171	0.3926	0.4888	0.779	0.5698	0	1.4921	1.6781	PREDICTED: transcription factor RAX3-like [Citrus sinensis]
Unigene00355	35.3372	2.8063	4.4446	10.5044	7.672	9.6472	1.8195	55.5707	14.4693	Cytochrome P450 [Theobroma cacao]
Unigene00358	11.2497	11.6635	2.1285	10.7338	5.9018	11.5256	2.2327	10.085	9.6041	PREDICTED: zinc finger protein ZAT4-like [Populus euphratica]
Unigene00359	52.9089	50.271	14.0834	50.9039	8.4569	58.5829	16.9509	46.6144	37.6423	NAC domain-containing 68 [Gossypium arboreum]
Unigene00359	39.4752	25.6743	16.3246	34.8384	5.5316	35.9482	39.0684	10.7983	7.2217	Transcription factor bHLH93-like protein [Gossypium arboreum]
Unigene00359	56.8447	96.5851	62.0357	134.9293	88.8803	116.0353	42.8181	165.2263	156.193	PREDICTED: transcription factor MYB1R1-like [Citrus sinensis]
Unigene00359	2.8994	5.2923	1.3492	2.347	0.6527	3.2166	5.0532	1.4064	0.4474	PREDICTED: MADS-box transcription factor 29-like isoform X1 [Citrus sinensis]
Unigene00361	1.3658	3.5486	3.6972	6.031	1.8551	5.6004	4.0394	3.5761	0.7926	PREDICTED: transcription factor TCP13 [Vitis vinifera]
Unigene00361	2.7	2.8942	7.9493	7.0317	8.2855	3.8984	1.6811	10.1326	1.9441	bZIP transcription factor family protein [Populus trichocarpa]
Unigene00361	17.1857	12.2025	53.4144	8.5147	17.8184	14.7486	83.1334	4.1554	13.162	PREDICTED: transcription factor WER [Vitis vinifera]

Unigene00361[illegible]	26.597	31.4063	4.6235	23.7463	27.3968	23.2422	9.9353	18.6769	18.9798	SOC1-1 [Litchi chinensis]
Unigene00365[illegible]	248.1839	253.316	32.4407	190.9318	16.177	152.3272	49.7856	544.4046	399.3574	PREDICTED: ethylene-responsive transcription factor ERF105-like [Citrus sinensis]
Unigene00365[illegible]	0.136	1.6457	0	1.1644	1.6069	1.6866	0.0335	1.8081	0.4405	--
Unigene00366[illegible]	8.98	25.4486	3.6256	28.9573	10.9356	17.9394	1.3896	80.7163	11.467	SAUR family protein [Theobroma cacao]
Unigene00368[illegible]	11.8904	6.3238	2.5449	5.431	1.6863	4.1445	10.4404	4.5935	8.8699	PREDICTED: auxin-induced protein 10A5 [Fragaria vesca subsp. vesca]
Unigene00368[illegible]	7.0871	22.3784	0.3818	15.0943	4.6966	13.2538	0.1625	11.5653	6.1316	abscisic acid 8’-hydroxylase 3-like precursor [Citrus sinensis]
Unigene00369[illegible]	3.1794	2.764	0.8149	1.5961	0.4928	3.911	1.9012	1.9172	3.1244	PREDICTED: heat stress transcription factor A-3-like isoform X1 [Citrus sinensis]
Unigene00370[illegible]	3.6913	2.0855	3.8222	0.9389	0.9245	2.0414	9.7364	0.3873	1.5842	PREDICTED: WUSCHEL-related homeobox 1 [Vitis vinifera]
Unigene00370[illegible]	3.9679	19.9222	1.3333	18.6167	6.8491	14.5329	1.3382	19.2632	6.7385	PREDICTED: transcription factor MYB3-like [Jatropha curcas]
Unigene00370[illegible]	10.181	44.4226	1.9568	42.9244	15.2753	28.3718	5.9319	34.214	49.8337	Gibberellin 2-beta-dioxygenase isoform 1 [Theobroma cacao]
Unigene00371[illegible]	5.8382	0.7599	0.0982	0.7331	0	1.8234	0.1392	2.771	3.8139	AP2/ERF domain transcription factor [Medicago truncatula]
Unigene00376[illegible]	13.6799	12.8329	1.1743	8.2142	0.2536	10.3882	5.4209	8.5003	11.1539	PREDICTED: auxin-induced protein 22D-like [Jatropha curcas]
Unigene00378[illegible]	4.7047	3.322	0.9808	2.4267	0.7124	4.9822	3.696	0.8985	0.5399	PREDICTED: B3 domain-containing transcription factor VRN1-like [Citrus sinensis]
Unigene00378[illegible]	272.9096	82.6458	87.9306	79.8154	115.4619	76.1067	52.7743	112.2536	186.733	PREDICTED: LOW QUALITY PROTEIN: acetyl-CoA acetyltransferase, cytosolic 1-like [Eucalyptus grandis]
Unigene00379[illegible]	55.7003	15.4318	0.2335	21.7158	0.3972	27.6901	18.1935	18.1295	62.8523	CRT/DRE-binding factor [Mangifera indica]
Unigene00380[illegible]	9.8966	6.0761	1.3833	5.7075	8.918	9.1323	2.1868	7.1025	5.4264	PREDICTED: probable transcription factor KAN4-like [Citrus sinensis]

Unigene003807	0	0.0898	0	0.5662	1.1972	0.7544	0	1.852	1.1783	putrescine aminopropyltransferase (spermidine synthase) [Chondrus crispus]
Unigene003811	3.3104	6.665	7.1926	4.2834	2.9756	3.8653	7.9059	1.8997	2.8201	PREDICTED: GATA transcription factor 21 isoform X2 [Vitis vinifera]
Unigene003843	1.0147	0.9553	0.4231	3.3883	1.1593	2.4561	0.5335	1.8184	1.5526	OPC-8:0 CoA ligase1 isoform 1 [Theobroma cacao]
Unigene003850	23.8653	14.4524	0.5994	20.7148	0.1359	15.1028	1.9842	37.5544	44.4097	PREDICTED: ethylene-responsive transcription factor ERF023-like [Citrus sinensis]
Unigene003857	0.4737	1.0376	0	2.7537	2.0154	3.1531	0	1.1258	0.5116	3-hydroxybutyryl-CoA dehydrogenase [Auxenochlorella protothecoides]
Unigene003882	225.6793	284.1082	267.9592	413.6738	634.0942	356.6289	144.4168	560.2253	557.8988	CONSTANS-like 4 [Theobroma cacao]
Unigene003883	0.7745	1.324	0	1.5526	3.5985	2.6506	0.3686	1.5719	0.3462	PREDICTED: 3-ketoacyl-CoA thiolase 2, peroxisomal-like isoform X2 [Glycine max]
Unigene003893	2.3841	4.9562	2.6683	3.1646	0.7621	2.7198	1.8494	2.3665	0.7809	PREDICTED: transcription factor bHLH130-like [Citrus sinensis]
Unigene003895	19.4275	34.2883	34.1372	37.3281	9.1105	28.9591	23.8088	29.3255	7.6762	PREDICTED: basic leucine zipper 9-like [Populus euphratica]
Unigene003898	52.7127	94.1953	25.819	86.6975	117.2915	82.7962	26.5355	77.8555	91.5601	Transcription factor APETALA2 isoform 1 [Theobroma cacao]
Unigene003902	25.5243	18.8676	1.9375	14.0483	2.17	32.208	19.7326	10.3648	53.0061	PREDICTED: ethylene-responsive transcription factor ERF027 [Jatropha curcas]
Unigene003908	6.7526	10.5846	6.3757	7.0493	3.081	7.0027	19.9584	0.7916	2.3487	violaxanthin de-epoxidase [Citrus sinensis]
Unigene003913	7.2519	5.5512	11.7466	3.478	6.1024	5.0905	18.0447	1.7564	2.7571	PREDICTED: transcription factor E2FA-like [Gossypium raimondii]
Unigene003918	2.6604	8.8916	2.1116	5.6502	4.7885	6.2329	1.179	9.8619	9.4113	PREDICTED: RING-H2 finger protein ATL52-like [Populus euphratica]

Unigene00394	3.6718	8.1794	2.2158	4.786	1.3867	5.6132	5.9604	1.9529	1.0734	PREDICTED: ethylene-responsive transcription factor CRF6-like [Citrus sinensis]
Unigene00396	33.999	58.9357	25.0205	104.6765	67.1241	94.3917	20.197	73.5282	72.9182	Aldehyde dehydrogenase family 2 member B4, mitochondrial [Gossypium arboreum]
Unigene00398	8.5405	8.7645	9.6716	4.06	4.2922	6.4722	26.2422	2.8456	3.3406	PREDICTED: cytochrome P450 85A [Jatropha curcas]
Unigene00399	28.5878	24.7176	10.4846	21.227	66.0095	26.5097	7.1763	18.9489	44.6	Plant-specific transcription factor YABBY family protein isoform 1 [Theobroma cacao]
Unigene00399	25.2462	24.8635	4.593	20.7102	39.4888	23.7293	4.9817	14.2524	27.0276	PREDICTED: axial regulator YABBY 5 isoform X1 [Vitis vinifera]
Unigene00401	0.9817	3.5366	1.2023	1.6815	0.7832	1.2178	2.4921	1.5555	0.3711	PREDICTED: homeobox-leucine zipper protein HAT5-like isoform X1 [Citrus sinensis]
Unigene00404	55.5779	65.3223	21.8111	77.3158	31.9304	58.3256	15.6722	115.2267	85.0172	PREDICTED: GATA transcription factor 5-like [Citrus sinensis]
Unigene00405	0.7801	1.4133	0	1.0605	2.5026	1.6411	0.0366	1.9551	0.5617	--
Unigene00406	14.7716	20.5369	22.7025	11.2307	12.5249	13.6442	32.3554	9.3452	11.3479	DIV1A protein [Theobroma cacao]
Unigene00406	0.4668	1.5731	0	1.6029	1.9946	1.3038	0	1.3412	0.7757	2-oxoglutarate dehydrogenase E1 component [Galdieria sulphuraria]
Unigene00407	28.0843	49.8849	12.1543	58.6725	62.1693	48.4011	16.0501	54.2918	47.7486	Floral homeotic protein APETALA 2 [Morus notabilis]
Unigene00409	0.4803	1.0152	0	1.4682	1.1354	2.0347	0	1.8725	0.5764	PREDICTED: agamous-like MADS-box protein AGL61 [Prunus mume]
Unigene00410	21.5931	45.7301	11.036	47.0825	55.5404	44.4501	16.0606	53.3328	39.0264	PREDICTED: abscisic acid receptor PYL9-like [Populus euphratica]
Unigene00411	51.3525	43.6771	11.3191	30.2614	9.3433	44.2644	28.9193	23.554	36.1874	PREDICTED: NAC domain-containing protein 69-like isoform X2 [Citrus sinensis]
Unigene00414	3.5828	5.8909	21.4684	7.9263	29.5946	4.8811	12.6036	4.055	10.2558	PREDICTED: malate dehydrogenase, glyoxysomal [Vitis vinifera]

Unigene004150	10.7145	13.9905	9.5727	10.243	4.7056	15.4815	15.3053	8.3628	4.379	PREDICTED: protein FAR-RED IMPAIRED RESPONSE 1 [Prunus mume]
Unigene004160	5.2097	3.7623	4.0884	3.3723	2.0524	4.294	4.7523	3.2023	1.8595	PREDICTED: prolycopene isomerase, chloroplastic isoform X1 [Citrus sinensis]
Unigene004183	0.0585	0	27.1549	0	0	0.3185	18.5344	0.3971	0.1263	K-box region and MADS-box transcription factor family protein isoform 3 [Theobroma cacao]
Unigene004188	60.0296	7.6058	24.2531	6.3415	4.4923	16.3638	17.2106	11.5232	11.5831	PREDICTED: allene oxide cyclase 4, chloroplastic-like [Citrus sinensis]
Unigene004194	11.6721	16.4776	14.1447	11.7011	9.1406	14.1732	19.386	6.4883	6.903	zeta carotene desaturase [Citrus maxima]
Unigene004198	6.0314	0.314	0	0.9291	3.5871	1.6954	0.4987	2.7745	4.9593	p-hydroxyphenylpyruvate dioxygenase [Mangifera indica]
Unigene004209	9.1733	19.2018	0.5157	7.9605	4.1417	9.8796	13.616	2.9864	1.9816	PREDICTED: transcription factor RAX2-like [Populus euphratica]
Unigene004210	6.3117	8.7734	0.5415	3.667	0.8288	2.892	4.0714	0.8231	1.2624	PREDICTED: transcription factor RAX2 [Populus euphratica]
Unigene004220	50.1691	61.6055	9.6131	30.7547	5.7416	39.7195	31.594	65.3163	41.7265	WRKY transcription factor 6-1 [Dimocarpus longan]
Unigene004273	0.1746	0.5033	1.4561	1.5407	2.3733	0.4227	0.8607	0.9881	0.0943	SERINE ACETYLTRANSFERASE-106 family protein [Populus trichocarpa]
Unigene004283	6.1038	4.0722	8.7886	2.8942	4.4947	4.7501	11.1409	2.1751	3.8737	PREDICTED: BEL1-like homeodomain protein 11 [Vitis vinifera]
Unigene004285	76.6983	54.2019	38.8252	44.2546	78.39	48.8841	38.3536	50.3583	109.5109	BES1/BZR1 [Theobroma cacao]
Unigene004294	4.6544	2.0498	2.5075	2.1172	0.8653	4.0507	5.6712	0.5919	0.3389	PREDICTED: transcription factor bHLH36 [Vitis vinifera]
Unigene004300	124.6079	298.1056	43.828	630.7072	377.3788	613.9119	24.5424	1369.2676	244.8201	PREDICTED: zinc finger CCCH domain-containing protein 20 [Jatropha curcas]
Unigene004308	0.7964	7.6761	0	1.7682	0.0523	0.1606	0.0654	0	0.0478	Aldehyde dehydrogenase family 2 member [Morus notabilis]
Unigene004319	10.0689	15.8045	8.3632	11.2487	25.8291	15.4426	12.543	12.3192	19.3176	PREDICTED: myb-like protein X [Gossypium raimondii]

Unigene00434	0.4595	1.9202	1.5765	1.3316	1.697	0.3244	1.5288	0.7367	0.9305	Nine-cis-epoxycarotenoid dioxygenase 4 [Theobroma cacao]
Unigene00434	98.7489	85.4518	4.4322	52.9476	113.8778	67.3255	12.8607	63.2639	98.2064	PREDICTED: MADS-box protein JOINTLESS [Populus euphratica]
Unigene00437	3.6515	7.5818	7.1706	9.1936	3.028	5.9574	9.1685	2.2604	2.898	PREDICTED: auxin-responsive protein IAA9-like [Jatropha curcas]
Unigene00438	0.9626	0.3551	2.0268	0.7394	0.1593	2.0271	0.3796	4.031	0.104	PREDICTED: protein ABSCISIC ACID-INSENSITIVE 5-like isoform X2 [Gossypium raimondii]
Unigene00439	5.4251	2.0061	0.1067	1.1157	1.0283	2.075	0.0505	1.2454	1.8795	PREDICTED: gibberellin 2-beta-dioxygenase 2-like [Citrus sinensis]
Unigene00440	0.4527	0.2236	0	1.2083	3.3245	2.7	0	1.354	0.0698	--
Unigene00443	2.3174	0.935	0	2.6462	0	0.4207	0	4.5899	0.876	CAP, cysteine-rich secretory protein, antigen 5, partial [Medicago truncatula]
Unigene00443	0.5265	0.3237	0	0.583	0	1.5718	0.0692	19.8628	5.4591	PREDICTED: pathogenesis-related protein PRB1-2-like [Beta vulgaris subsp. vulgaris]
Unigene00445	17.9364	66.4607	9.683	75.4263	28.0578	73.8677	8.0739	105.4461	31.8733	PREDICTED: heat shock factor protein HSF30-like [Citrus sinensis]
Unigene00446	4.9489	6.583	1.5872	1.8277	4.3867	4.4921	13.0416	1.885	0.2056	PREDICTED: tetraspanin-19-like isoform X2 [Gossypium raimondii]
Unigene00447	3.0669	7.955	0.3475	13.8593	0.2167	10.9536	2.054	19.9396	3.5645	abscisic acid 8'-hydroxylase 1-like [Citrus sinensis]
Unigene00447	357.1551	8.4941	19.6294	10.6175	40.4177	8.2132	4.492	74.9692	125.3121	Salicylate O-methyltransferase [Gossypium arboreum]
Unigene00449	1.8824	0.3756	0	1.8038	0	0.7886	0	10.079	1.2121	--
Unigene00450	8.1418	12.1445	1.8625	18.1456	11.6791	11.6777	1.976	23.3183	8.4245	GBF's pro-rich region-interacting factor 1 [Theobroma cacao]
Unigene00452	11.2304	15.311	23.4117	12.8762	15.1781	13.9368	34.1332	13.1945	11.3833	PREDICTED: transcription factor PIF3-like isoform X1 [Citrus sinensis]
Unigene00453	22.6717	37.8483	19.6538	37.2836	42.2275	31.1724	15.6652	34.1326	41.7789	C2H2-like zinc finger protein [Theobroma cacao]

Unigene004545	4.7691	26.3617	8.6249	31.7351	12.2041	18.6145	5.7061	25.3578	1.1153	PREDICTED: cyclic dof factor 3-like [Citrus sinensis]
Unigene004553	2.2321	2.2487	0.4821	1.4403	0.7605	1.9226	0.0396	2.5718	2.4325	PREDICTED: bifunctional L-3-cyanoalanine synthase/cysteine synthase D1-like isoform X1 [Gossypium raimondii]
Unigene004554	49.2451	20.6802	18.6263	21.7215	16.0433	18.9584	7.9706	65.0156	38.1546	HSF20 [Gossypium hirsutum]
Unigene004610	18.1568	31.7482	24.0547	19.3691	13.9905	29.0281	29.0753	12.093	11.8402	AP2 domain-containing transcription factor [Theobroma cacao]
Unigene004645	6.8554	15.5183	6.5382	9.4215	2.329	11.8239	16.8002	2.6701	2.5481	amidase family protein [Populus trichocarpa]
Unigene004655	2.2971	11.9013	1.0183	9.8396	3.5885	11.2799	0.9761	4.5272	2.1245	Regulatory protein isoform 1 [Theobroma cacao]
Unigene004668	1.6956	5.7754	0.2731	6.4007	3.3521	5.0558	1.2613	2.774	1.8813	VfN-28 family protein [Populus trichocarpa]
Unigene004674	0.3054	2.8633	0	2.1556	2.2377	5.2974	0.0803	2.5346	0.5497	O-acetylserine sulfhydrylase/homocysteine synthase [Ostreococcus lucimarinus CCE9901]
Unigene004709	1.9457	1.7901	0	3.3793	0	1.5397	0.0369	10.1213	1.3943	PREDICTED: similar to Zinc finger protein 271 (Zinc finger protein 7) (HZF7) (Zinc finger protein ZNFphex133) (Epstein-Barr virus-induced zinc finger protein) (ZNF-EB) (CT-ZFP48) (Zinc finger protein [Bathycoccus prasinos]
Unigene004722	22.5829	37.4792	1.6545	60.0378	44.699	49.493	4.1539	51.5754	41.7688	Zinc finger protein 4 [Theobroma cacao]
Unigene00473	0.7662	2.2483	0	3.2536	1.3582	6.9122	0	2.6014	0.3009	--
Unigene004735	18.2056	29.6587	35.928	26.7325	15.0557	29.8101	34.5453	20.7599	17.1617	PREDICTED: AP2-like ethylene-responsive transcription factor AIL6-like isoform X2 [Citrus sinensis]
Unigene004792	39.6568	33.0561	2.1564	20.1493	1.618	24.467	8.6379	48.1715	19.7649	WRKY transcription factor 17 [Dimocarpus longan]
Unigene004792	0.215	5.3279	1.1203	5.1323	5.208	5.3331	1.6953	3.5276	4.179	transcription factor NtWRKY4 [Nicotiana tabacum]
Unigene004793	3.794	1.8547	99.6677	2.9414	5.6476	2.0343	60.1765	1.6035	4.0978	PREDICTED: developmental protein SEPALLATA 1 [Nelumbo nucifera]

Unigene00481	1.1282	1.4518	0.0547	1.8792	1.3023	1.6829	0	1.5738	0.5101	putrescine aminopropyltransferase (spermidine synthase) [Chondrus crispus]
Unigene00481[illegible]	0.8066	1.3019	0	1.1053	1.8685	1.6791	0	4.5282	0.453	s-adenosylmethionine decarboxylase [Nannochloropsis gaditana]
Unigene00482	42.1249	14.9328	20.2806	10.7012	6.5063	16.9505	62.519	3.4818	6.9661	PREDICTED: myb-related protein Zm38 [Vitis vinifera]
Unigene00484[illegible]	4.8624	7.6339	8.2471	3.4094	5.2174	7.3948	11.3428	3.3671	5.1942	PREDICTED: AT-rich interactive domain-containing protein 1-like isoform X1 [Citrus sinensis]
Unigene00486[illegible]	2.0492	6.0301	1.1242	5.8778	8.3701	6.1783	3.7036	2.6854	3.3004	WRKY transcription factor 34 [Jatropha curcas]
Unigene00487[illegible]	32.345	24.6725	38.396	31.4306	77.9628	53.5463	24.7042	80.6871	2.8798	late elongated hypocotyl a [Dimocarpus longan]
Unigene00489[illegible]	28.7611	83.3319	22.8711	49.7682	28.4286	66.4764	72.7466	25.7638	147.4179	gigantea [Dimocarpus longan]
Unigene00490[illegible]	0.8928	1.6099	0	1.2952	1.3393	1.4961	0	2.9796	3.1402	vitamine B12-independent methionine synthase [Cyanidioschyzon merolae strain 10D]
Unigene00491[illegible]	17.9226	17.5293	26.0615	17.8322	11.6872	13.4858	24.2368	16.9433	12.5289	PREDICTED: transcription factor BIM1 isoform X1 [Jatropha curcas]
Unigene00492[illegible]	1.1218	1.1084	3.6748	0.7486	1.1364	0.4849	3.0806	0.9975	0.7789	PREDICTED: two-component response regulator ARR8-like [Jatropha curcas]
Unigene00495[illegible]	2.4594	1.4858	3.3154	1.1415	1.1459	2.2754	7.915	0.4282	0.9706	ethylene response 2 precursor [Citrus sinensis]
Unigene00495[illegible]	37.6808	63.8697	51.5824	113.0643	76.6998	93.689	35.2115	120.7808	25.1678	ABA responsive element binding factor [Citrus trifoliata]
Unigene00500[illegible]	5.0366	11.6127	1.1413	6.3272	2.0273	10.2477	5.9368	1.6936	0.473	short vegetative phase 2 [Dimocarpus longan]
Unigene00500[illegible]	43.9558	5.2241	4.1995	4.7383	1.8696	24.6795	3.9232	14.3791	17.068	PREDICTED: linoleate 13S-lipoxygenase 3-1, chloroplastic-like [Citrus sinensis]
Unigene00502[illegible]	0	0.4352	0	0.4355	0	0	0.0827	0	0	PREDICTED: B3 domain-containing transcription factor VRN1-like [Citrus sinensis]

Unigene00503	16.7756	22.381	10.9184	19.7241	6.539	20.8033	22.206	6.8859	4.7424	Transcription factor IIIA [Gossypium arboreum]
Unigene00503	4.5636	8.6752	5.634	7.1398	2.2951	9.7537	7.3707	5.0369	2.1149	Transcription factor IIIA [Gossypium arboreum]
Unigene00504	34.6845	16.0235	4.4432	15.7902	1.421	13.2722	9.8953	12.3292	9.1097	PREDICTED: NAC domain-containing protein 89-like isoform X1 [Citrus sinensis]
Unigene00513	55.2918	50.8397	9.0665	41.2046	5.6541	35.0905	14.5777	52.7341	55.9299	PREDICTED: transcription factor PCL1-like [Tarenaya hassleriana]
Unigene00515	21.1572	5.7292	3.3971	5.8693	5.8097	9.022	2.4674	18.1986	13.0496	PREDICTED: heat shock factor protein HSF24-like [Citrus sinensis]
Unigene00515	0.342	0.0282	0	0.9636	0.0866	0.7982	0.3371	0.387	0.7651	PREDICTED: heat shock factor protein HSF24-like [Citrus sinensis]
Unigene00516	0	1.7139	0	2.5544	1.8782	1.9854	0	5.5119	0.5537	Aldehyde dehydrogenase family 2 member [Morus notabilis]
Unigene00516	9.2071	1.0798	0.4985	17.2536	0	5.2097	0.1572	4.6689	0	PREDICTED: aldehyde dehydrogenase family 2 member B4, mitochondrial-like [Brassica rapa]
Unigene00516	3.2817	5.3554	2.7691	3.6278	0.4197	3.7482	12.8148	1.3024	1.1508	PREDICTED: B3 domain-containing transcription factor VRN1-like [Citrus sinensis]
Unigene00529	6.1266	0.8643	0.0447	1.2231	0.3038	0.7	0.0845	2.0608	3.3549	PREDICTED: transcription factor bHLH101-like [Nelumbo nucifera]
Unigene00532	30.0862	26.3282	1.2279	19.0146	0.8529	17.3006	1.9146	26.7846	18.1446	1-aminocyclopropane-1-carboxylate synthase-like [Citrus sinensis]
Unigene00532	1.7966	3.5411	0.0242	3.5737	3.1919	5.457	0.1143	5.5361	1.2269	vitamine B12-independent methionine synthase [Cyanidioschyzon merolae strain 10D]
Unigene00534	13.3405	14.6366	28.4476	11.9575	8.7695	12.7175	25.2521	4.6013	4.6549	Alanine--glyoxylate aminotransferase 2 isoform 3 [Theobroma cacao]
Unigene00535	1.3536	0.5749	0	2.5145	0	1.0778	0	6.0877	1.9237	malate dehydrogenase [Coccomyxa subellipsoidea C-169]
Unigene00535	3.6171	3.6671	0	4.5804	8.7317	5.1228	0	8.7683	3.408	malate dehydrogenase [Sargassum fusiforme]

Unigene00539[illegible]	8.3074	7.6447	10.0148	6.3418	4.3949	6.8616	15.612	3.141	4.3923	PREDICTED: CRC domain-containing protein TSO1-like isoform X3 [Citrus sinensis]
Unigene00544	2.5481	0.9792	0.0192	2.2427	0	1.095	0	2.5078	1.1368	S-adenosylmethionine synthetase [Galdieria sulphuraria]
Unigene00544[illegible]	44.2267	23.047	18.3531	14.9404	4.4721	17.5773	51.1261	4.6788	5.5753	PREDICTED: 12-oxophytodienoate reductase 2-like isoform X1 [Citrus sinensis]
Unigene00546[illegible]	7.3249	6.3333	3.0672	4.1166	5.0449	4.1421	1.0458	6.0176	11.5807	PREDICTED: primary amine oxidase-like [Citrus sinensis]
Unigene00549[illegible]	49.3149	12.7362	72.8191	6.5489	17.7364	16.1772	112.4076	8.4695	30.7551	DNA (cytosine-5)-methyltransferase CMT3-like [Citrus sinensis]
Unigene00552[illegible]	0.2272	1.7285	0	0.8961	1.2888	1.0448	0	3.1368	3.1898	S-adenosyl-L-homocysteine hydrolase-like protein, partial [Eleusine coracana subsp. coracana]
Unigene00552[illegible]	0	0.5565	0	1.3959	1.3855	1.2101	0	3.5901	1.9364	PREDICTED: LOW QUALITY PROTEIN: adenosylhomocysteinase-like [Beta vulgaris subsp. vulgaris]
Unigene00552[illegible]	3.1851	0.9442	0	4.4401	0	1.1564	0.0897	3.6049	2.9487	S-adenosyl-L-homocysteine hydrolase-like protein, partial [Eleusine coracana subsp. coracana]
Unigene00557[illegible]	1.0964	2.8138	0	3.9564	2.4681	6.0659	0.0257	3.8113	0.6485	--
Unigene00557[illegible]	1.4556	6.8918	0.0232	7.9938	6.1461	8.432	0.0438	7.9105	2.1363	PREDICTED: similar to Zinc finger protein 271 (Zinc finger protein 7) (HZF7) (Zinc finger protein ZNFphex133) (Epstein-Barr virus-induced zinc finger protein) (ZNF-EB) (CT-ZFP48) (Zinc finger protein [Bathycoccus prasinos]
Unigene00557[illegible]	0.4266	0.843	0.3176	0.5377	2.3046	0.6637	0.4205	0.6897	1.3821	PREDICTED: ethylene-responsive transcription factor ERF113-like isoform X1 [Citrus sinensis]

Unigene00560	15.8023	3.6607	15.9262	1.5254	3.8853	4.7172	24.3253	2.1704	6.5634	PREDICTED: DNA (cytosine-5)-methyltransferase 1B-like [Cucumis melo]
Unigene00562	1.1907	0.2422	2.0442	0.3635	0.9934	1.8649	0.4834	3.5673	2.0426	amidase [Cucumis melo subsp. melo]
Unigene00562	1.0306	1.246	2.5414	0.7567	1.4854	1.7036	3.0727	0.7112	0.8416	zeatin O-glucosyltransferase-like protein [Medicago truncatula]
Unigene00564	0	1.2348	0.0558	1.0563	0.7595	0.1296	0.3168	0	0.0578	amidase family protein [Populus trichocarpa]
Unigene00566	1.3572	1.695	0.0196	4.6474	2.2055	5.4296	0.0372	2.8588	0.8551	--
Unigene00566	0.1517	0.3497	0.0226	0.4836	0.2049	0.1311	0.1282	0.0736	0.2106	ethylene receptor [Dimocarpus longan]
Unigene00567	0	2.6263	0.5397	4.4073	2.3253	2.5065	0.1021	1.4064	2.0132	PREDICTED: auxin response factor 1 [Fragaria vesca subsp. vesca]
Unigene00571	9.6311	42.2766	7.7222	61.5802	120.2052	32.9189	6.9906	24.5557	38.8084	pathogenesis-related gene 1-like protein PR1-2, partial [Dimocarpus longan]
Unigene00573	0.6869	0.198	0	1.9907	0	0.6581	0	1.976	0.8037	--
Unigene00574	17.554	7.3697	5.2226	3.5871	6.1259	5.8549	1.6352	25.0689	17.616	ethylene response factor 1-like protein ERF1-1 [Dimocarpus longan]
Unigene00575	1.4842	3.4649	0.5028	2.8504	9.2531	2.2903	0.2561	3.3177	2.2844	PREDICTED: allene oxide synthase-like [Vitis vinifera]
Unigene00579	19.4378	1.4153	0.2844	10.7616	21.6831	7.5114	1.6809	6.0981	13.5542	PREDICTED: transcription factor RADIALIS-like [Fragaria vesca subsp. vesca]
Unigene00581	12.1982	12.0871	15.7339	5.0397	4.5323	4.74	12.1462	7.3416	3.1285	R2R3 MYB C2 repressor 2 protein [Theobroma cacao]
Unigene00589	8.9189	4.8675	2.2747	3.9398	4.3734	3.445	1.5902	3.4901	8.762	Mads box-like protein [Theobroma cacao]
Unigene00596	0	0.0762	3.7897	0	0	0	4.3012	0	0	K-box region and MADS-box transcription factor family protein isoform 1 [Theobroma cacao]
Unigene00596	28.0821	1.0117	0.5047	0.4712	0.1431	1.941	0.0597	5.6168	3.4318	PREDICTED: myb-related protein Myb4-like [Populus euphratica]
Unigene00597	0.7057	0.4407	2.1455	0.4884	0.556	0.605	1.3624	0.3328	0.5717	NAC domain containing protein 20 [Theobroma cacao]

Unigene005982	29.9286	30.2525	8.1745	32.3557	23.5678	26.365	8.5244	53.5215	25.9542	PREDICTED: auxin-induced protein 15A-like [Jatropha curcas]
Unigene005993	3.5989	2.1485	13.2633	0.8006	1.557	1.9444	12.4494	1.9638	3.297	PREDICTED: ethylene-responsive transcription factor ERF084-like [Populus euphratica]
Unigene005995	14.4834	32.1081	21.803	32.5783	9.1417	32.5484	23.6094	9.6606	6.5167	PREDICTED: 4-coumarate--CoA ligase-like 7-like [Citrus sinensis]
Unigene006009	0.2361	2.1777	0.9845	1.2256	0	0	0.2328	0.5345	0.255	auxin response factor 1 family protein [Populus trichocarpa]
Unigene006009	0	1.6	0.4133	1.1938	0.3281	0.3839	0	3.1417	1.1992	SAUR family protein [Theobroma cacao]
Unigene006030	0.1453	1.8427	0	0.1508	0.3435	1.6707	0	0.1645	0	enolase-phosphatase E1 [Monoraphidium neglectum]
Unigene00604[illegible]	1.4306	1.0527	0	0.316	0.2158	0.921	0.8702	0.5856	0.4603	PREDICTED: transcription factor bHLH18-like [Gossypium raimondii]
Unigene006062	7.4919	4.3731	0.3954	2.7565	0.523	1.4538	1.2465	0.8587	2.2534	PREDICTED: ethylene-responsive transcription factor ERF014-like [Citrus sinensis]
Unigene006100	0.2594	1.1961	16.1766	2.7374	1.9415	1.2557	0	0	1.0272	PREDICTED: transcription factor ILI6-like [Musa acuminata subsp. malaccensis]
Unigene006134	0.1498	0.1728	0	1.4778	0.1771	0	0	1.7808	0	PYR1-like 11 [Theobroma cacao]
Unigene006147	7.8559	0.7833	2.6557	0.7053	0.4015	1.1306	4.0607	2.8356	0.0917	PREDICTED: NAC transcription factor NAM-B2-like [Citrus sinensis]
Unigene006159	4.3469	3.6006	8.9276	1.8384	1.2939	2.3382	5.5551	3.9358	1.5302	MYB-related transcription factor [Salvia miltiorrhiza]
Unigene006202	7.0042	0.5383	1.4297	0.1212	0.2759	0.4238	1.6112	0.3964	2.963	PREDICTED: transcription factor HEC2-like [Citrus sinensis]
Unigene00631[illegible]	0.1699	0.1958	0	1.4988	0	0	0	1.0573	0.0917	PREDICTED: cytochrome P450 CYP82D47-like [Solanum lycopersicum]
Unigene006369	0	1.2806	4.7677	0.5427	3.7069	4.6659	0	0.4437	0.9881	PREDICTED: pathogenesis-related protein 1-like [Citrus sinensis]

Unigene006373	9.0766	3.0602	5.5337	5.4222	9.1081	3.2644	4.3896	4.6518	13.0403	Integrase-type DNA-binding superfamily protein [Theobroma cacao]
Unigene006440	0	0	0	0.7786	0.8865	0	0	2.4524	0	cysteine dioxygenase [Nannochloropsis gaditana]
Unigene006448	15.1384	32.3064	20.0117	27.093	32.9814	34.4841	8.4454	46.9909	45.1985	PREDICTED: ethylene-responsive transcription factor RAP2-10-like [Citrus sinensis]
Unigene006509	0	0.417	0.3142	1.9401	0.9263	0.7296	0.1189	1.7741	1.0418	Myb domain protein 79 isoform 1 [Theobroma cacao]
Unigene00652	7.7922	2.1137	5.1758	2.2998	1.8057	2.3114	13.255	2.8532	1.4852	PREDICTED: transcription factor CPC-like [Jatropha curcas]
Unigene006668	0.2716	0.0783	0	1.3746	0	0.0411	0	2.6899	0.6601	zinc finger protein Gfi-1 [Bathycoccus prasinos]
Unigene006677	4.3322	13.6061	5.3944	14.0994	6.6146	10.4863	2.2624	24.5424	19.1718	PREDICTED: protein SENSITIVE TO PROTON RHIZOTOXICITY 1-like [Populus euphratica]
Unigene006688	1.8783	6.7319	8.9169	4.4402	2.4177	3.4211	8.7819	1.0945	4.2181	Protein EARLY FLOWERING 3 -like protein [Gossypium arboreum]
Unigene006705	0	2.2085	0.8095	0.5912	0	0.376	0.4083	1.6408	0.0559	FRIGIDA [Dimocarpus longan]
Unigene006708	0.7817	0	0	1.1157	0	0	0	0.7741	0.6332	PREDICTED: zinc finger protein 347-like [Bathycoccus prasinos]
Unigene006714	7.474	5.4018	1.5195	5.9691	8.382	5.4749	0.9695	4.6223	6.398	PREDICTED: AP2-like ethylene-responsive transcription factor BBM2 [Populus euphratica]
Unigene006723	0.2738	2.0518	1.5221	0.758	0	1.1046	2.4744	0	0.4929	PREDICTED: probable salt tolerance-like protein At1g78600-like [Citrus sinensis]
Unigene006750	0.2135	2.1535	0.445	0.4986	0.1262	0.7428	0.9734	0.2416	0	PREDICTED: NAC domain-containing protein 18-like [Citrus sinensis]
Unigene006769	20.8941	11.5354	19.8607	13.0139	37.6526	28.3033	4.3464	35.9706	32.8362	PREDICTED: ethylene-responsive transcription factor 2-like [Populus euphratica]
Unigene006775	1.0439	0.4298	0.7772	0.4644	0.3525	0.6317	1.2497	0.5064	2.255	4-coumarate:CoA ligase 3 [Epimedium sagittatum]

Unigene00680	0.3979	0.4587	0	0.1377	1.5675	0.4816	0.3923	1.5011	1.3609	PREDICTED: cytokinin dehydrogenase 3-like [Citrus sinensis]
Unigene00690	4.2143	11.8782	4.1597	4.2498	1.5857	6.7034	6.6305	0.9734	1.3191	PREDICTED: NAC domain-containing protein 8-like [Citrus sinensis]
Unigene00690	92.447	18.002	1.4069	16.4689	0.6381	18.17	13.6748	45.4997	106.802	PREDICTED: ethylene-responsive transcription factor ERF109-like [Jatropha curcas]
Unigene00691	4.7895	6.201	0.6656	3.5951	5.4283	4.8453	0	4.8647	6.3403	Gibberellin 3-beta-dioxygenase 1 [Morus notabilis]
Unigene00693	0.0723	0	3.882	0	0	0	5.953	0	0	PREDICTED: MADS-box transcription factor 23-like isoform X2 [Vitis vinifera]
Unigene00694	0	2.1103	1.3408	0.6163	1.7541	1.9161	0	4.3116	0.9618	PREDICTED: ethylene-responsive transcription factor RAP2-1-like [Citrus sinensis]
Unigene00695	0.0781	0.1801	0	0.6082	0.7386	0.0945	0	1.1936	1.2234	PREDICTED: 12-oxophytodienoate reductase 2-like [Populus euphratica]
Unigene00697	0.0344	0.8329	0	1.0714	1.8297	0.1665	0.0678	1.7132	1.189	PREDICTED: beta-carotene isomerase D27, chloroplastic-like [Malus domestica]
Unigene00698	4.5914	3.4948	1.1658	3.5442	0.8348	2.1729	0.2902	3.7975	2.6703	PREDICTED: AP2/ERF and B3 domain-containing transcription repressor RAV2-like [Citrus sinensis]
Unigene00711	0	0	0.1041	0	1.4168	0.2418	0	0.4523	1.7264	7-methylxanthosine synthase 1 [Morus notabilis]
Unigene00715	2.5974	0.2303	0.0417	3.6089	0	1.209	0	2.8492	0	aspartate aminotransferase [Sargassum hemiphyllum var. chinense]
Unigene00716	3.0381	2.3998	0.2932	2.745	0.1995	1.2256	1.9413	2.8336	2.7649	PREDICTED: zinc finger protein 3-like [Vitis vinifera]
Unigene00723	0.2319	1.8568	1.0073	0.8025	0.67	0.7797	6.0337	0.1167	0.4732	PREDICTED: protein TRANSPARENT TESTA 1-like [Nicotiana sylvestris]
Unigene00724	9.362	8.292	2.4991	6.3408	5.9708	5.7343	2.8701	4.2965	6.9363	auxin-regulated protein [Phaseolus vulgaris]

Unigene00726[illegible]	0.4974	0.1274	0.6336	0.2295	0	0.0669	0.2179	0.0625	0.2388	PREDICTED: probable transcription factor KAN4 isoform X3 [Gossypium raimondii]
